# Supplementary figures and images for: Comparative chloroplast genomes: insights into the evolution of the chloroplast genome of Camellia sinensis and the phylogeny of Camellia
Source: BMC Genomics. 2021 Feb 26;22:138. doi: 10.1186/s12864-021-07427-2 (PMC7912895; doi:10.1186/s12864-021-07427-2)

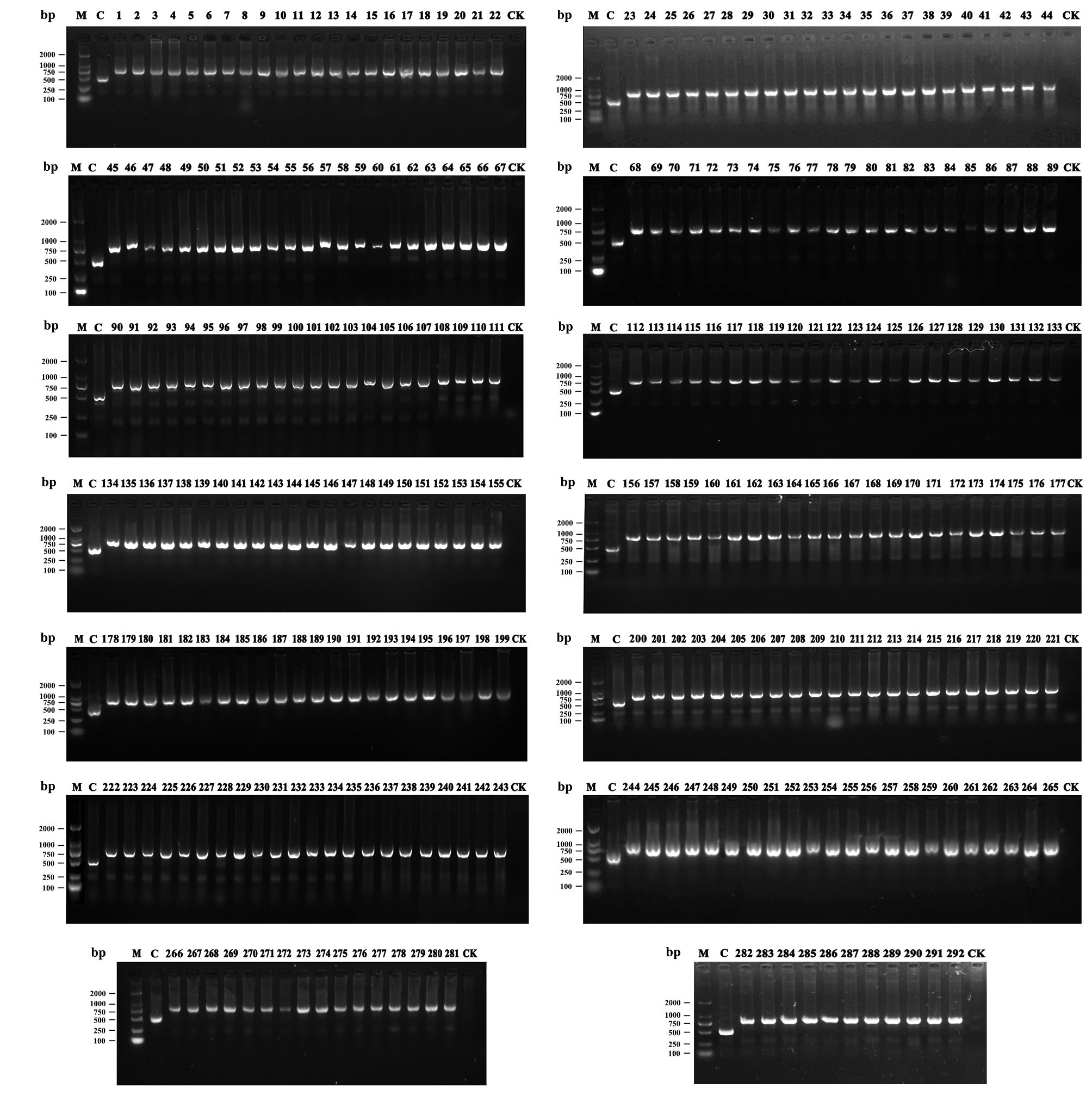

Supplement: Supplementary file 1 — Additional file 1: Supplementary Fig. S1. SCAR analysis was conducted by PCR amplification of the respective chloroplast region in 292 different cultivars of Camellia sinensis. PCR amplification to screen the distribution of a 335 bp deletion of the intergenic spacers (trnE/trnT) in 292 different cultivars of Camellia sinensis. None of 292 cultivars had this sequence deletion like triploid CWN. M: D2000 DNA molecular marker; C: PCR products of CWN; Lane 1–292: PCR products of 292 different cultivars covering the majority of C. sinensis cultivars in China. CK: Control. The corresponding 292 cultivars were shown in Supplementary Tab. S5. [file 12864_2021_7427_MOESM1_ESM.jpg]

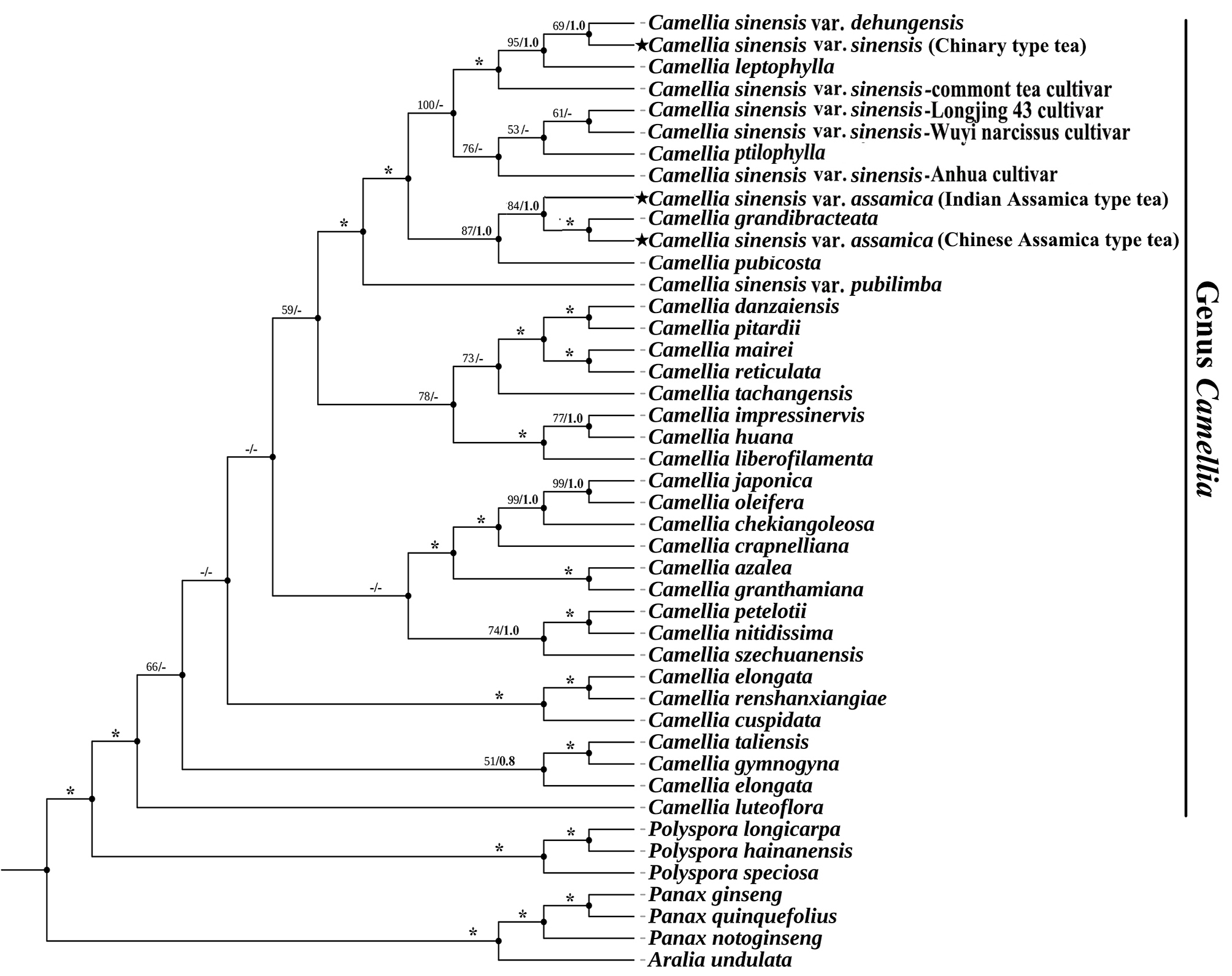

Supplement: Supplementary file 2 — Additional file 2: Supplementary Fig. S2. Phylogeny of Camellia inferred from maximum likelihood (ML) analysis of LSC. Numbers associated with nodes indicated ML bootstrap support (BS)/Bayesian inference (BI) posterior probabilities (PPs) values. Asterisks represented nodes with maximal support values in both analyses. Dash denoted nodes unresolved or with BS/PPs support in the ML/BI trees less than 50%/0.5. C. sinensis var. sinensis (Chinary type tea) and two C. sinensis var. assamica (Chinese Assamica type tea and Indian Assamica type tea) were highlighted with star mark. [file 12864_2021_7427_MOESM2_ESM.jpg]

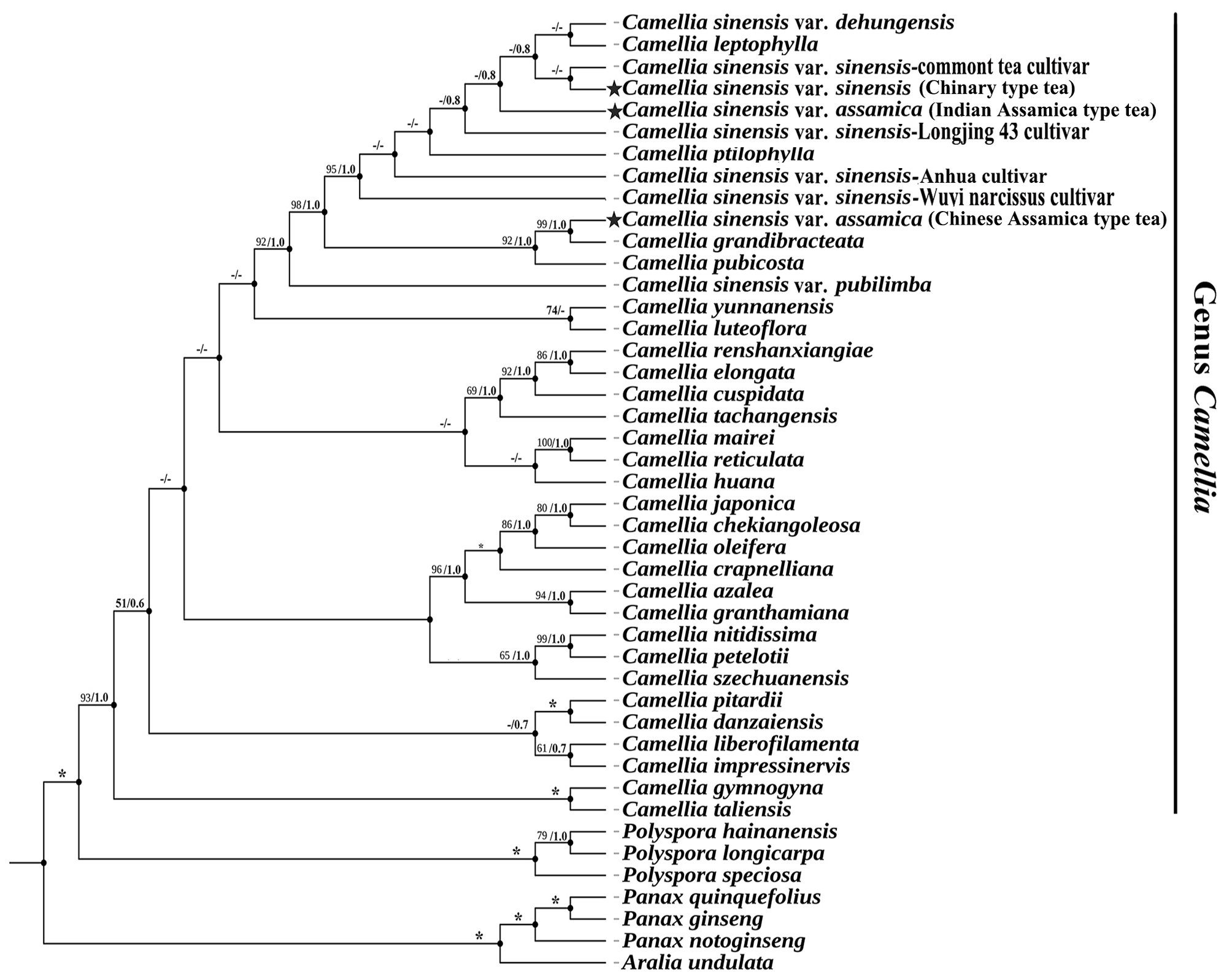

Supplement: Supplementary file 3 — Additional file 3: Supplementary Fig. S3. Phylogeny of Camellia inferred from maximum likelihood (ML) analysis of SSC. The meaning of the figures and symbols was consistent with Fig. S2. [file 12864_2021_7427_MOESM3_ESM.jpg]

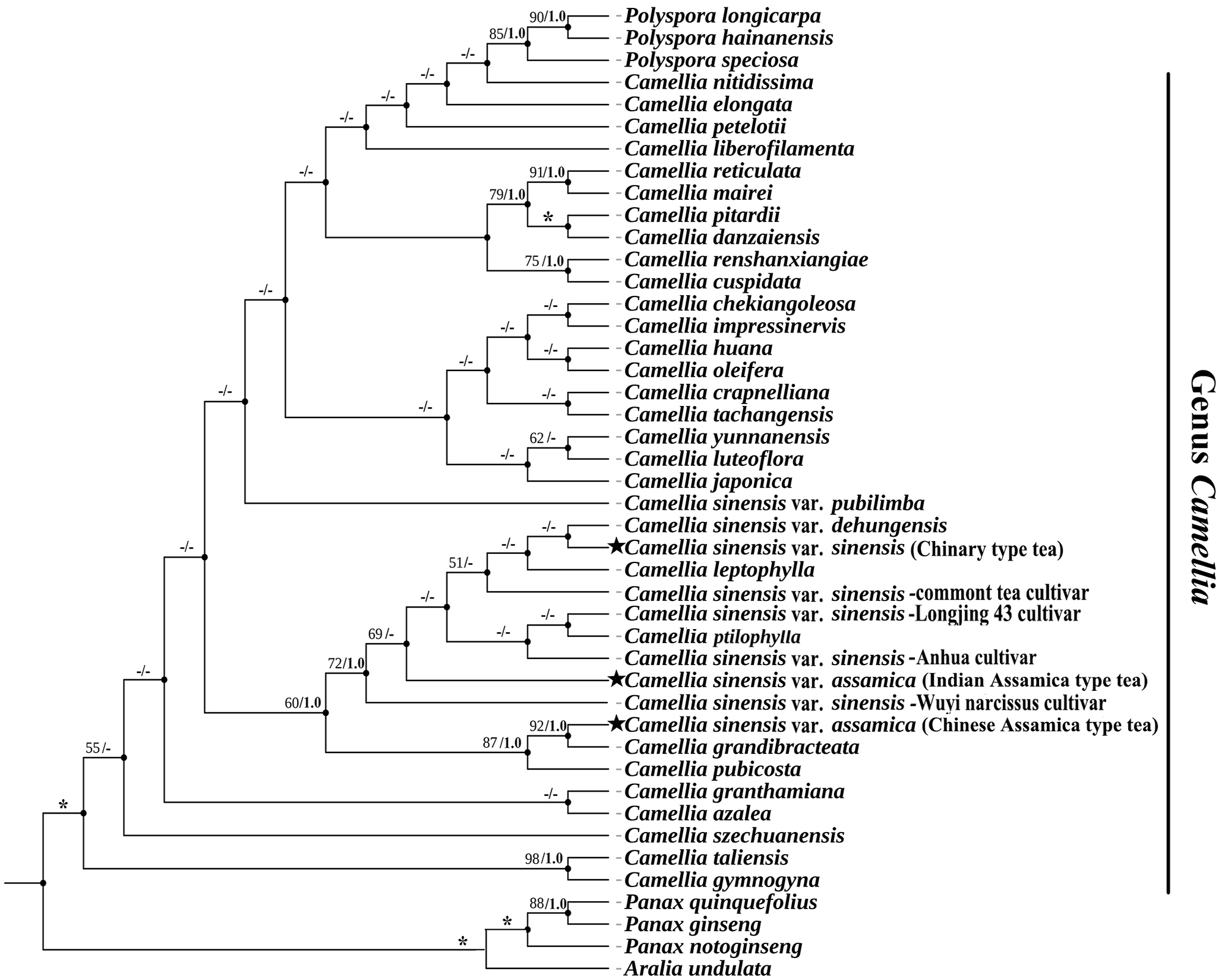

Supplement: Supplementary file 4 — Additional file 4: Supplementary Fig. S4. Phylogeny of Camellia inferred from maximum likelihood (ML) analysis of IR. The meaning of the figures and symbols was consistent with Fig. S2. [file 12864_2021_7427_MOESM4_ESM.jpg]

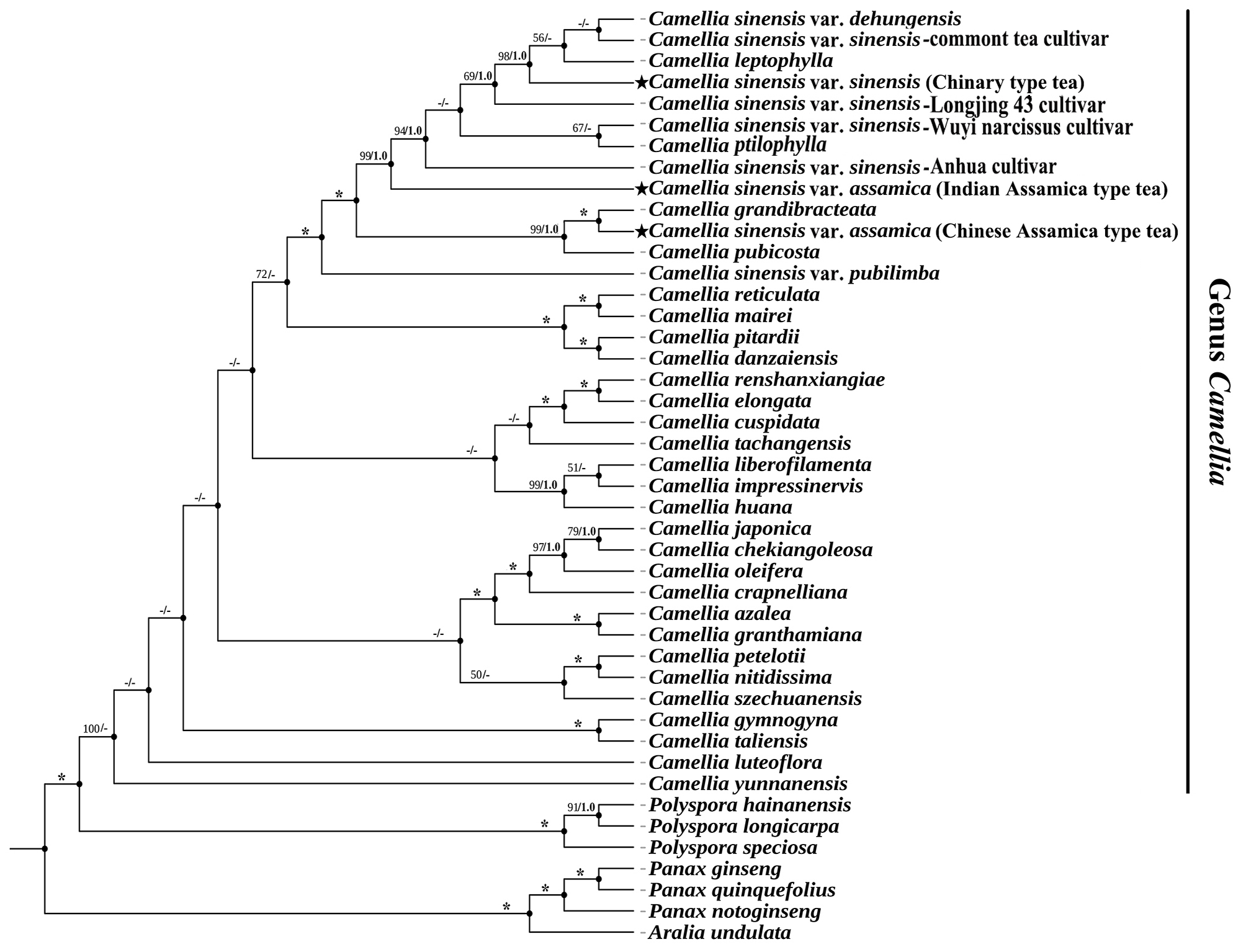

Supplement: Supplementary file 5 — Additional file 5: Supplementary Fig. S5. Phylogeny of Camellia inferred from maximum likelihood (ML) analysis of PCGs. The meaning of the figures and symbols was consistent with Fig. S2. [file 12864_2021_7427_MOESM5_ESM.jpg]

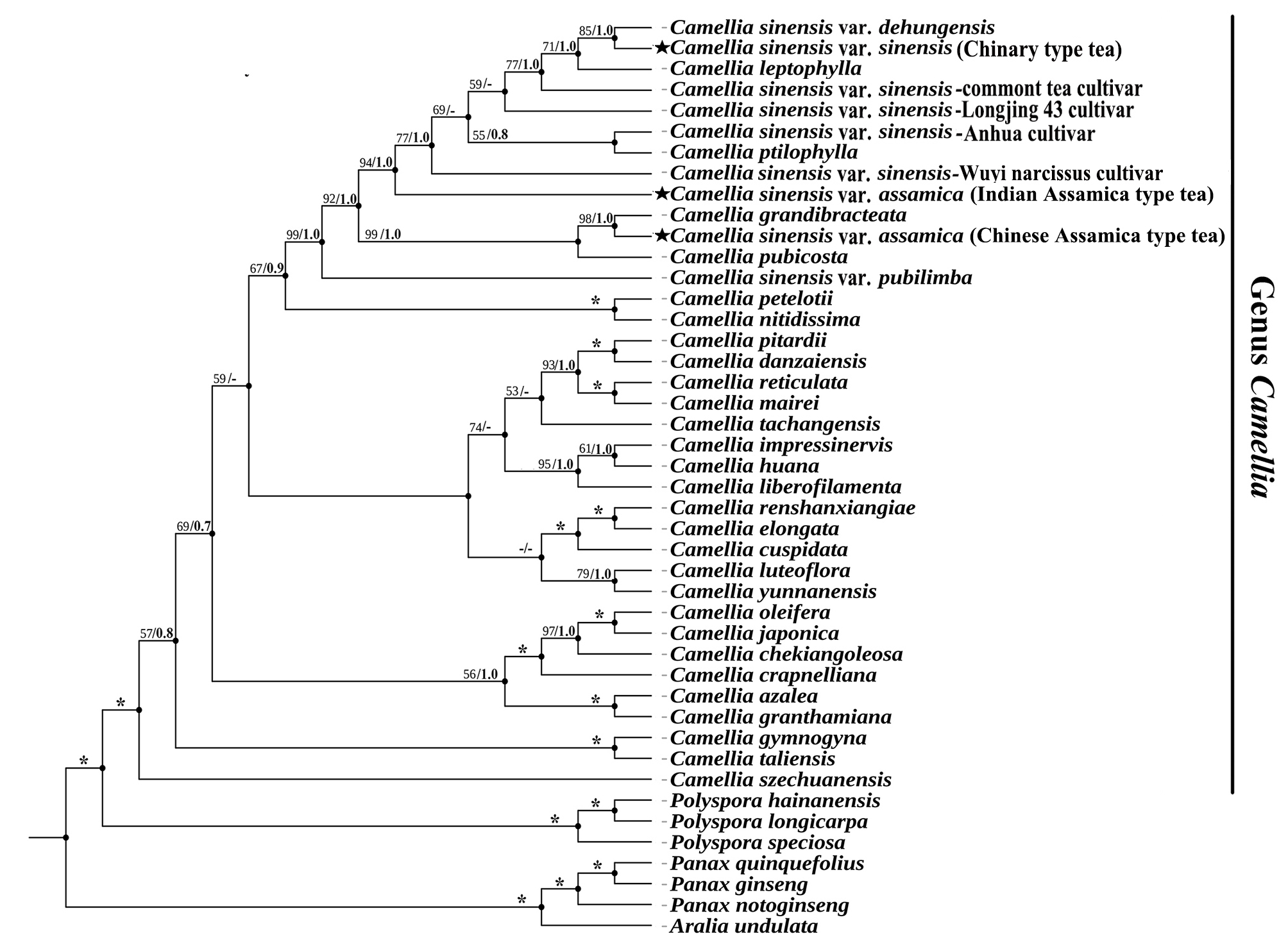

Supplement: Supplementary file 6 — Additional file 6: Supplementary Fig. S6. Phylogeny of Camellia inferred from maximum likelihood (ML) analysis of Non-PCGs. The meaning of the figures and symbols was consistent with Fig. S2. [file 12864_2021_7427_MOESM6_ESM.jpg]
