# Supplementary material for: Comparative chloroplast genomes: insights into the evolution of the chloroplast genome of Camellia sinensis and the phylogeny of Camellia
Source: BMC Genomics. 2021 Feb 26;22:138. doi: 10.1186/s12864-021-07427-2 (PMC7912895; doi:10.1186/s12864-021-07427-2)
Supplement: Supplementary file 7 — Additional file 7: Supplementary Tab. S1. List of genes in the cp genome. * Genes containing a single introns; ** Genes containing two introns. [file 12864_2021_7427_MOESM7_ESM.doc]

**Supplementary Tab. S1 List of genes in cp genome.**

| **category** | **group** | **genes** |
| --- | --- | --- |
| Photosynthesis related genes | Rubisco | rbcL |
|  | Photosystem I | psaA, psaB, psaC, psaI, psaJ |
|  | Assembly/stability of Photosystem I | ycf3**, ycf4 |
|  | Photosystem II | psbA, psbB, psbC, psbD, psbE, psbF, psbH, psbI, psbJ, psbK, psbL, psbM, psbN, psbT, psbZ |
|  | ATP synthase | atpA, atpB, atpE, atpF*, atpH, atpI |
|  | Cytochrome b/f complex | petA, petB*, petD*, petG, petL, petN |
|  | Cytochrome c synthesis | ccsA |
|  | NADPH dehydrogenase | ndhA*, ndhB* (2x), ndhC, ndhD, ndhE, ndhF, ndhG, ndhH, ndhI, ndhJ, ndhK |
|  | acetyl-CoA carboxylase | accD |
| Transcription related genes | RNA polymerase | rpoA, rpoB, rpoC1*, rpoC2 |
|  | Ribosomal proteins (SSU) | rps2, rps3, rps4, rps7 (2x), rps8, rps11, rps12* (2x), rps14, rps15, rps16*, rps18, rps19. |
|  | Ribosomal proteins (LSU) | rpl2* (2x), rpl14, rpl16*, rpl20, rpl22, rpl23 (2x), rpl32, rpl33, rpl36 |
|  | RNA processing | matK |
| RNA genes | Ribosomal RNA | rrn5S (2x), rrn4.5 (2x), rrn16S (2x), rrn23S (2x) |
|  | Transfer RNA | trnH-GUG, trnK-UUU*, trnQ-UUG, trnS-GCU, trnG-GCC* (2x), trnR-UCU, trnC-GCA, trnD-GUC, trnY-GUA, trnE-UUC, trnT-GGU, trnS-UGA, trnM-CAU (2x), trnS-GGA, trnT-UGU, trnL-UAA*, trnF-GAA, trnV-UAC*, trnW-CCA, trnP-UGG, trnI-GAU* (2x), trnL-CAA (2x), trnV-GAC (2x), trnA-UGC* (2x), trnR-ACG (2x), trnN-GUU (2x), trnL-UAG, trnI-CAU(2x) |
| Other genes | membrane protein | cemA |
|  | Proteolysis | clpP** |
|  | Translation initiation factor | infA |
| Genes of unknown function | Conserved ORFs | ycf1 (2x), ycf2 (2x), ycf15 (4x), orf42 (2x) |

* Genes containing a single introns; ** Genes containing two introns.
