# Supplementary material for: Comparative chloroplast genomes: insights into the evolution of the chloroplast genome of Camellia sinensis and the phylogeny of Camellia
Source: BMC Genomics. 2021 Feb 26;22:138. doi: 10.1186/s12864-021-07427-2 (PMC7912895; doi:10.1186/s12864-021-07427-2)
Supplement: Supplementary file 8 — Additional file 8: Supplementary Tab. S2. Distribution of the different and the shared SSRs in four Camellia chloroplast genomes. CWN: ‘Wuyi narcissus’ cultivar of C. sinensis var. sinensis (natural triploid Chinary type tea); CSS: C. sinensis var. sinensis (diploid Chinary type tea); CSA: C. sinensis var. assamica (diploid Chinese Assamica type tea); CIA: C. sinensis var. assamica (diploid Indian Assamica type tea). Symbol “/” indicated missing. [file 12864_2021_7427_MOESM8_ESM.docx]

**Supplementary Table S2-1.** **Distribution of the different simple sequence repeats (SSRs) in the four *Camellia* chloroplast genomes**

| **No.** | **Location** | **Locus** | **SSR type** | | | | **Region** |
| --- | --- | --- | --- | --- | --- | --- | --- |
|  |  |  | ***CSS*** | ***CSA*** | ***CIA*** | ***CWN*** |  |
| **1** | IGS | rpl2/trnH-GTG | / | / | (A)8 | / | LSC |
| **2** | IGS | matK/rps16 | (A)11 | (A)9 | (A)9 | (A)10 |  |
| **3** | IGS | matK/rps16 | (A)9taagaattgattctgaacataaaaatgaacagatccgatgaaaatacaagaaattatc(A)9tatatagataaatatcaaaattgatagaccccatc(A)8 | (A)9taagaattgattctgaacataaaaatgaacagatccgatgaaaatacaagaaattatc(A)9 | (A)9taagaattgattctgaacataaaaatgaacagatccgatgaaaatacaagaaattatc(A)9 | (A)9taagaattgattctgaacataaaaatgaacagatccgatgaaaatacaagaaattatc(A)9tatatagataaatatcaaaattgatagaccccatc(A)8 |  |
| **4** | Intron | rps16 | (A)10 | (A)10 | (A)10 | (A)9 |  |
| **5** | IGS | rps16/trnQ-UUG | (T)9 | (T)8cttctatatagatgtttttctgaagaagttttagcaagaattcaacttaaccccctattttagtgtataaatattttattgtataaatagaaatgaaa(T)8 | (T)8cttctatatagatgtttttctgaagaagttttagcaagaattcaacttaaccccctattttagtgtataaatattttattgtataaatagaaatgaaa(T)9 | (T)10 |  |
| **6** | IGS | psbI/trnS-GCT | (A)12 | (A)12 | (A)12 | (A)11 |  |
| **7** | IGS | trnS-GCU/trnG-GCC | (A)9tacttgctattaaagcaacaagaaagtgtcttttcttattattcctttcttccttttttatttgc(T)8aactactttactggaaactggaat(A)8 | / | (A)9tacttgctattaaagcaacaagaaagtgtcttttcttattattcctttcttccttttttatttgc(T)9aactactttactggaaactggaat(A)8 | (A)9tacttgctattaaagcaacaagaaagtgtcttttcttattattcctttcttccttttttatttgc(T)8aactactttactggaaactggaat(A)8 |  |
| **8** | IGS | trnS-GCU/trnG-UCC | / | (T)10atcccatggccgggctaggtcagtatctagccgggcctttttttgttccaacgaatcatagtatctgattg(A)9tacttgctattaaagcaacaagaaagtgtcttttcttattattcctttcttccttttttatttgc(T)9aactactttactggaaactggaat(A)8 | / | / |  |
| **9** | Intron | trnG-UCC | / | (T)8 | (T)8 | / |  |
| **10** | IGS | atpA/atpF | (A)16 | (A)16 | (A)16 | (A)18 |  |
| **11** | IGS | atpH/atpI | (T)11 | (T)14 | (T)11 | (T)12 |  |
| **12** | IGS | atpH/atpI | (T)13 | (T)12 | (T)12 | (T)12 |  |
| **13** | IGS | trnC-GCA/petN | / | (T)8 | / | / |  |
| **14** | IGS | trnE-TTC/trnT-GGT | (A)13 | (A)13 | (A)13 | / |  |
| **15** | IGS | trnE-TTC/trnT-GGT | (A)11 | (A)10 | (A)11 | (A)11 |  |
| **16** | IGS | trnT-GTT/psbD | (T)17 | (T)16 | (T)13 | (T)12 |  |
| **17** | IGS | psbC/trnS-TGA | (T)10 | (T)9 | (T)9 | (T)10 |  |
| **18** | IGS | trnS-TGA/trnG-GCC | (A)13 | (A)13 | (A)15 | (A)13 |  |
| **19** | IGS | psaA/ycf3 | (A)9 | (A)10 | / | (A)9 |  |
| **20** | CDS | ycf3 | (A)11 | (A)12 | (A)11 | (A)11 |  |
| **21** | IGS | trnT-TGT/trnF-GAA | (T)10 | (T)9 | (T)10 | (T)9 |  |
| **22** | IGS | ndhK/ndhC | (T)11 | (T)11 | (T)11 | (T)12 |  |
| **23** | IGS | ndhC/trnV-UAC | (T)9 | (T)9 | (T)8 | (T)8 |  |
| **24** | IGS | ndhC/trnV-UAC | (T)16atagggctatacggactcgaaccgtagaccttctcggtaaaacagatcaaactgattattatcaaaatgatccgaactgtttcaaagacccaacgtgca(T)9 | / | (T)16atagggctatacggactcgaaccgtagaccttctcggtaaaacagatcaaactgattattatcaaaatgatccgaactgtttcaaagacccaacgtgca(T)9 | (T)16 |  |
| **25** | IGS | ndhC/trnM-CAU | / | (T)11 | / | (T)9 |  |
| **26** | Intron | trnV-UAC | / | (T)9 | / | / |  |
| **27** | IGS | atpB/rbcL | (T)8cagtatatcaatttatacctagcctaattctttcttttcg(T)21 | (T)10cagtatatcaatttatacctagcctaattctttcttttcg(T)14 | (T)9cagtcatatcaatttatacctagcctaattc(T)9g(T)14 | (T)8cagtatatcaatttatacctagcctaattctttcttttcg(T)13 |  |
| **28** | IGS | rbcL/accD | (T)11 | (T)14 | (T)11 | (T)11 |  |
| **29** | IGS | accD/psaI | (T)8ggtgacataagatctaattgtagaaagaatcaaaagttgcggataattc(T)15 | (T)8ggtgacataagatctaattgtagaaagaatcaaaagttgcggataattc(T)15 | (T)8ggtgacataagatctaattgtagaaagaatcaaaagttgcggataattc(T)15 | (T)8ggtgacataagatctaattgtagaaagaatcaaaagttgcggataattc(T)14 |  |
| **30** | IGS | ycf4/cemA | (T)9 | (T)10 | (T)9 | (T)9 |  |
| **31** | IGS | petA/psbJ | (A)10 | (A)10 | (A)11 | (A)10 |  |
| **32** | IGS | petA/psbJ | (T)11cagtttttacatataatatataataaaagggtagtggacaaacaaaaaaagatagggaaacttattgagtaaataaaacttcttcaatgaacttat(A)8ttcaaatttgatgagatactaataaatagagtaaagttctattactatagaaagtatttgcatgatatctaatctacagaaata(AT)4 | (A)9ttcaaatttgatgagatactaataaatagagtaaagttctattactatagaaagtatttgcatgatatctaatctacagaaata(AT)4 | (T)11cattttttacatataatatataataaaagggtagtggacaaacaaaaaaagatagggaaacttattgagtaaataaaacttcttcaatgaacttat(A)8ttcaaatttgatgagatactaataaatagagtaaagttctattactatagaaagtatttgcatgatatctaatctacagaaata(AT)4 | (T)10cagtttttacatataatatataataaaaaggtagtggacaaacaaaaaaagatagggaaacttattgagtaaataaaacttcttcaatgaacttat(A)8ttcaaatttgatgagatactaataaatagagtaaagttctattactatagaaagtatttgcatgatatctaatctacagaaata(AT)4 |  |
| **33** | IGS | rps18/rpl20 | (T)10 | (T)11 | (T)10 | (T)10 |  |
| **34** | Intron | clpP | (T)9gag(A)9 | (T)9gag(A)11 | (T)9gag(A)9 | (T)9gag(A)11 |  |
| **35** | Intron | clpP | (A)9 | (A)8 | (A)9 | (A)8 |  |
| **36** | CDS | rps8 | (A)10 | / | (A)8 | (A)10 |  |
| **37** | IGS | rps8/rpl14 | (T)16 | (T)16 | (T)15 | (T)15 |  |
| **38** | IGS | rpl14/rpl16 | (T)8aatctgttctttcaatgcaaggggcgaag(A)10 | (T)8aatctgttctttcaatgcaaggggcgaag(A)12 | (T)8aatctgttctttcaatgcaaggggcgaag(A)10 | (T)8aatctgttctttcaatgcaaggggcgaag(A)12 |  |
| **39** | Intron | trnI-GAU | (T)10 | (T)11 | (T)10 | (T)10 | IRb |
| **40** | IGS | rrn5S/rrn4.5S | (CCCT)3 | / | (CCCT)3 | (CCCT)3 |  |
| **41** | IGS | ndhF/rpl32 | (T)12aaat(A)9 | (T)12aaat(A)8 | (T)12 | (T)13aaat(A)9 | SSC |
| **42** | CDS | rpl32 | / | / | / | (A)9 |  |
| **43** | IGS | ccsA/ndhD | (T)12aaat(A)9 | (T)9 | / | (T)9 |  |
| **44** | CDS | ndhD | (AATA)3 | (AATA)3 | / | (AATA)3 |  |
| **45** | Intron | ndhA | (T)8 | (T)8 | (T)8 | / |  |
| **46** | IGS | rrn4.5S/rrn5S | (GAGG)3 | / | (GAGG)3 | (GAGG)3 | IRa |
| **47** | Intron | trnI-GAU | (A)10 | (A)11 | (A)10 | (A)10 |  |
| **Total** | **159** |  | **40** | **40** | **39** | **40** |  |
|  | **Number of different locations: CDS, 11; IGS, 122; Intron, 26** | | | | | |  |

*CWN*: ‘Wuyi narcissus’ cultivar of *C. sinensis* var. *sinensis* (natural triploid Chinary type tea); *CSS*: *C. sinensis* var. *sinensis* (diploid Chinary type tea); *CSA*: *C. sinensis* var. *assamica* (diploid Chinese Assamica type tea); *CIA*: *C. sinensis* var. *assamica* (diploid Indian Assamica type tea). Symbol “/” indicated missing.

**Supplementary Table S2-2. Distribution of the shared simple sequence repeats (SSRs) in the four Camellia chloroplast genomes.**

| **No.** | **Location** | **Locus** | **SSR type** | **Region** |
| --- | --- | --- | --- | --- |
| **1** | IGS | trnH-GTG/psbA | (A)13 | LSC |
| **2** | CDS | matK | (T)8gaggatccgctatgataatgagaaagatttc(TA)4 |  |
| **3** | IGS | matK/rps16 | (AT)4tttcgaaaaaaatacaatggaaataatatt(A)8ggacttgtgttggattggcactccatctataatctatatagatacaaaaaatggaaatagaggaataaataaggaagaagtag(A)8 |  |
| **4** | IGS | matK/rps16 | (A)9 |  |
| **5** | IGS | rps16/trnQ-UUG | (AGAT)3 |  |
| **6** | IGS | rps16/trnQ-TTG | (T)9 |  |
| **7** | CDS | psbK | (T)8 |  |
| **8** | IGS | psbK/psbI | (T)12 |  |
| **9** | IGS | trnS-GCU/trnG-UCC | (A)8gataattactatgttacattacacgtaaagtaaggattcaaaaaagtctttctt(TC)4 |  |
| **10** | Intron | trnG-GCC | (T)9 |  |
| **11** | IGS | trnR-TCT/atpA | (C)8 |  |
| **12** | CDS | atpA | (GTCT)3 |  |
| **13** | CDS | atpF | (T)8 |  |
| **14** | CDS | atpF | (A)8 |  |
| **15** | IGS | atpF/atpH | (T)12 |  |
| **16** | IGS | atpI/rps2 | (T)8 |  |
| **17** | IGS | rps2/rpoC2 | (A)11 |  |
| **18** | CDS | rpoC2 | (T)8 |  |
| **19** | CDS | rpoC2 | (T)9 |  |
| **20** | CDS | rpoC2 | (T)9cctattcctggtggtatcaagatgccactgtgtcgggatatcttatctgtctctccaggaaaatggagatctccagaaaagattttaagctcaattc(T)11 |  |
| **21** | CDS | rpoC2 | (A)8 |  |
| **22** | CDS | rpoC2 | (T)9accagaaaaatccgaactaaaaaatttgtgcctcacttgatcattagtcaatgagggattagaa(AT)4 |  |
| **23** | CDS | rpoC2 | (CT)4 |  |
| **24** | CDS | rpoC2 | (AT)5 |  |
| **25** | CDS | rpoC1 | (AT)4 |  |
| **26** | CDS | rpoC1 | (A)8 |  |
| **27** | CDS | rpoB | (T)10 |  |
| **28** | IGS | rpoB/trnC-GCA | (T)8 |  |
| **29** | IGS | rpoB/trnC-GCA | (A)8 |  |
| **30** | IGS | rpoB/trnC-GCA | (A)9 |  |
| **31** | IGS | trnC-GCA/petN | (A)9 |  |
| **32** | IGS | petN/psbM | (GA)4 |  |
| **33** | IGS | psbM/trnD-GTC | (T)8 |  |
| **34** | IGS | psbM/trnD-GTC | (AG)4 |  |
| **35** | IGS | trnE-TTC/trnT-GGT | (T)9 |  |
| **36** | IGS | trnT-GTT/psbD | (T)9 |  |
| **37** | IGS | trnT-GTT/psbD | (T)9atttaatattgtattgaatttaatgaaaaaatagaaaattctctttttttctaacagataaaaataaatcgaaaaatattcgaagta(TCTT)3cgacccgtggaaagatatactttggagttttagattcatctgaaggaaaaggaaacataacaaagaagacactaaaagaaaatg(A)10tagaaataaaaaaagtaaattaat(A)8 |  |
| **38** | CDS | trnS-TGA | (GA)4 |  |
| **39** | IGS | trnS-TGA/trnG-GCC | (A)8ttagaggggggtcaaactatttaaatgaaaaaaattaataaaatagttggaatcgccctgaagagagtctctggcccggcactacacaaatataatcc(AT)4catatatgtgtggacatattgcgtatcaagaacg(A)11 |  |
| **40** | IGS | trnG-GCC/trnM-CAT | (A)11 |  |
| **41** | CDS | ycf3 | (TTTC)3 |  |
| **42** | IGS | ycf3/trnS-GGA | (A)13 |  |
| **43** | IGS | rps4/trnT-TGT | (TA)4 |  |
| **44** | IGS | trnT-TGT/trnF-GAA | (T)10 |  |
| **45** | IGS | trnT-TGT/trnF-GAA | (A)12gaatcgaccgttcaagtattccaaattacatgagaaaaattataaaaggagaaag(AT)4 |  |
| **46** | IGS | trnF-GAA/ndhJ | (T)8 |  |
| **47** | CDS | atpB | (T)10 |  |
| **48** | IGS | atpB/rbcL | (AT)4 |  |
| **49** | IGS | accD/psaI | (TA)4 |  |
| **50** | IGS | psaI/ycf4 | (T)9 |  |
| **51** | CDS | ycf4 | (T)8 |  |
| **52** | IGS | ycf4/cemA | (AAAT)3 |  |
| **53** | CDS | cemA | (TC)4 |  |
| **54** | CDS | petA | (AT)4 |  |
| **55** | CDS | petA | (A)8 |  |
| **56** | IGS | petA/psbJ | (T)10 |  |
| **57** | IGS | trnP-TGG/psaJ | (T)9 |  |
| **58** | IGS | psaJ/rpl33 | (T)8 |  |
| **59** | IGS | psaJ/rpl33 | (TTC)4gtttcggatcaaaaatagaagagttgagtaaat(A)8 |  |
| **60** | IGS | rpl33/rps18 | (TA)4 |  |
| **61** | IGS | rpl20/rps12 | (A)8 |  |
| **62** | Intron | clpP(intron) | (A)8 |  |
| **63** | Intron | clpP(intron) | (T)8 |  |
| **64** | IGS | psbB/psbT | (A)8 |  |
| **65** | IGS | psbH/petB | (A)8 |  |
| **66** | IGS | petB/petD | (AT)4 |  |
| **67** | IGS | petD/rpoA | (T)8 |  |
| **68** | CDS | rpoA | (TA)4ttggatcttttgaggcaattatagattctgggaggcaattctaattggtcaataaaaatatatttcgatgcta(T)10 |  |
| **69** | IGS | infA/rps8 | (T)10 |  |
| **70** | IGS | rpl16/rps3 | (AT)5ttttatttatagataatattgtttttataacataacgaagc(T)8 |  |
| **71** | IGS | rpl16/rps3 | (T)8 |  |
| **72** | IGS | rpl16/rps3 | (T)12 |  |
| **73** | CDS | rpl22 | (AT)4 |  |
| **74** | IGS | rps19/rpl2 | (T)9 | IRA |
| **75** | CDS | rpl2 | (TA)5 |  |
| **76** | CDS | ycf2 | (GA)4tatt(GA)4 |  |
| **77** | CDS | ycf2 | (GA)4 |  |
| **78** | CDS | ycf2 | (A)9 |  |
| **79** | CDS | ycf2 | (A)9 |  |
| **80** | CDS | ycf2 | (TCTA)3 |  |
| **81** | CDS | ycf2 | (TA)4 |  |
| **82** | CDS | ndhB | (AG)4 |  |
| **83** | IGS | ndhB/rps7 | (TA)4 |  |
| **84** | IGS | rps7/trnV-GAC | (T)8 |  |
| **85** | IGS | rrn16S/trnI-GAU | (C)8 |  |
| **86** | Intron | trnI-GAU | (G)8 |  |
| **87** | CDS | rrn23S | (CT)4 |  |
| **88** | IGS | rrn4.5S/rrn5S | (A)11 |  |
| **89** | IGS | trnR-ACG/trnN-GTT | (CTTTTT)3 |  |
| **90** | CDS | ndhF | (A)8 | SSC |
| **91** | IGS | ndhF/rpl32 | (A)9ttatattcttaattgttttcgattcaccaaatcttatctctttcgaaagggttaat(A)8 |  |
| **92** | IGS | ndhF/rpl32 | (T)8 |  |
| **93** | IGS | rpl32/trnL-TAG | (A)8tacttttctttg(A)11taaacacaagatacaaaggtttacctttctttttagtcta(T)8 |  |
| **94** | IGS | rpl32/trnL-TAG | (AT)4 |  |
| **95** | IGS | trnL-UAG/ccsA | (T)9 |  |
| **96** | CDS | ccsA | (A)11 |  |
| **97** | CDS | ccsA | (T)8 |  |
| **98** | CDS | ndhD | (GAAA)3 |  |
| **99** | CDS | ndhD | (T)8 |  |
| **100** | IGS | psaC/ndhE | (T)8 |  |
| **101** | IGS | ndhG/ndhI | (AAAT)3cgaaatatcatgactttgttgacctgaccagtaaaaaagaagttactttc(T)14 |  |
| **102** | Intron | ndhA | (T)9aacaa(T)8 |  |
| **103** | Intron | ndhA | (A)9tgaaa(AT)4 |  |
| **104** | CDS | rps15 | (T)9 |  |
| **105** | CDS | ycf1 | (T)9 |  |
| **106** | CDS | ycf1 | (T)12cattcgagggtataacccccttttgctttctattgatgtttttattttcactaccattttcatttatatt(A)8 |  |
| **107** | CDS | ycf1 | (T)9 |  |
| **108** | CDS | ycf1 | (T)8attattgtaaatggatttatcaatca(T)8 |  |
| **109** | CDS | ycf1 | (T)9attttttgtaattctttttatttgatttctgattgtgcttgttctatcagtcagatctttca(T)9ctgtcagtaaaaaatttgtccaatatgtagattgaattcgactaaaggatttatgaattatatgattgcgggttatagaatctttttc(T)12agtttcgcttgatttatatatttctctcaatctaaataatagaattggatttacttttgagagttcttttttta(T)8aaaaacggaatgcccttgataatccatttttttg(T)8gagatatttagaaattttgttctttcttttaaaacttttagaaatataaaatac(T)8caattttccaa(T)9cgagtttcttaaaaatgggttcaaaaaaggaaggccgtttttggggagaaccaaaaggaagttcagcttccattccccaaaccgtt(A)12 |  |
| **110** | CDS | ycf1 | (A)10 |  |
| **111** | CDS | ycf1 | (T)17 |  |
| **112** | CDS | ycf1 | (T)8ccataactttgccgtgtat(A)8 |  |
| **113** | IGS | trnN-GTT/trnR-ACG | (AAAAAG)3 | IRB |
| **114** | IGS | trnR-ACG/rrn5S | (T)11 |  |
| **115** | CDS | rrn23S | (AG)4 |  |
| **116** | Intron | trnI-GAU | (C)8 |  |
| **117** | IGS | trnI-GAU/rrn16S | (G)8 |  |
| **118** | IGS | trnV-GAC/rps7 | (A)8 |  |
| **119** | IGS | rps7/ndhB | (TA)4 |  |
| **120** | CDS | ndhB | (CT)4 |  |
| **121** | CDS | ycf2 | (TA)4 |  |
| **122** | CDS | ycf2 | (ATAG)3 |  |
| **123** | CDS | ycf2 | (T)9 |  |
| **124** | CDS | ycf2 | (T)9 |  |
| **125** | CDS | ycf2 | (TC)4 |  |
| **126** | CDS | ycf2 | (TC)4aata(TC)4 |  |
| **127** | Intron | rpl2 | (AT)5 |  |
| **128** | IGS | rpl2/trnH-GUG | (A)9 |  |
| **Number of different locations: CDS, 55; IGS, 65; Intron, 8** | | | | |
