# Supplementary material for: Comparative chloroplast genomes: insights into the evolution of the chloroplast genome of Camellia sinensis and the phylogeny of Camellia
Source: BMC Genomics. 2021 Feb 26;22:138. doi: 10.1186/s12864-021-07427-2 (PMC7912895; doi:10.1186/s12864-021-07427-2)
Supplement: Supplementary file 9 — Additional file 9: Supplementary Tab. S3. A list of repeated sequences and their locations identified in four Camellia chloroplast genomes. CWN: ‘Wuyi narcissus’ cultivar of C. sinensis var. sinensis (natural triploid Chinary type tea); CSS: C. sinensis var. sinensis (diploid Chinary type tea); CSA: C. sinensis var. assamica (diploid Chinese Assamica type tea); CIA: C. sinensis var. assamica (diploid Indian Assamica type tea). [file 12864_2021_7427_MOESM9_ESM.docx]

**Supplementary Tab. S3 A list of repeated sequences and their locations identified in the four *Camellia* chloroplast genomes.**

| **Tandem repeats** | | | | | | | | | | | | | **Shared** |
| --- | --- | --- | --- | --- | --- | --- | --- | --- | --- | --- | --- | --- | --- |
| ***CSS*** | | | | ***CSA*** | | | ***CIA*** | | | ***CWN*** | | |  |
| **No.** | **Len** | **Position** | **Copy Num.** | **Len** | **Position** | **Copy Num.** | **Len** | **Position** | **Copy Num.** | **Len** | **Position** | **Copy Num.** |  |
| **1** | 15 | [129737-129792](#129737--129792,15,3.9,14,26)  ycf1(CDS) | 3.9 | 15 | [129721-129777](#129721--129777,14,3.9,15,22)  ycf1(CDS) | 3.9 | 15 | [130009-130063](#130009--130063,14,3.9,14,26)  ycf1(CDS) | 3.9 | 15 | [129382-129437](#129382--129437,15,3.9,14,26)  ycf1(CDS) | 3.9 | All |
| **2** | 16 | [6349-6382](#6349--6382,16,2.1,16,2)  rps16/trnQ-UUG  (IGS) | 2.1 | 16 | [6338-6371](#6338--6371,16,2.1,16,2)  rps16/trnQ-UUG  (IGS) | 2.1 | 16 | [6988-7021](#6988--7021,16,2.1,16,2)  rps16/trnQ-UUG  (IGS) | 2.1 | 16 | [6340-6373](#6340--6373,16,2.1,16,2)  rps16/trnQ-UUG  (IGS) | 2.1 | All |
| **3** | 16 | [48779-48809](#48779--48809,16,1.9,16,5)  trnT-UGU/trnL-UAA(IGS) | 1.9 | 16 | [48748-48778](#48748--48778,16,1.9,16,4)  trnT-UGU/trnL-UAA  (IGS) | 1.9 | 16 | [49415-49445](#49415--49445,16,1.9,16,5)  trnT-UGU/trnL-UAA(IGS) | 1.9 | 16 | [48402-48432](#48402--48432,16,1.9,16,4)  trnT-UGU/trnL-UAA  (IGS) | 1.9 | All |
| **4** | 16 | [53227-53257](#53227--53257,16,1.9,16,6)  ndhC/trnV-UAC  (IGS) | 1.9 | 16 | [53205-53235](#53205--53235,16,1.9,16,5)  ndhC/trnV-UAC  (IGS) | 1.9 | 16 | [53860-53891](#53860--53891,16,2.0,16,6)  ndhC/trnV-UAC  (IGS) | 2 | 16 | ndhC/trnV-UAC  (IGS) | 2 | All |
| **5** | 16 | [60774-60819](#60774--60819,16,2.9,16,7)  accD/psaI(IGS) | 2.9 | 16 | [60757-60802](#60757--60802,16,2.9,16,6)  accD/psaI(IGS) | 2.9 | 16 | [61412-61457](#61412--61457,16,2.9,16,8)  accD/psaI(IGS) | 2.9 | 16 | [60409-60454](#60409--60454,16,2.9,16,6)  accD/psaI(IGS) | 2.9 | All |
| **6** | 16 | [60777-60832](#60777--60832,16,3.5,16,9)  accD/psaI(IGS) | 3.5 | 16 | [60760-60815](#60760--60815,16,3.5,16,8)  accD/psaI(IGS) | 3.5 | 16 | [61415-61470](#61415--61470,16,3.5,16,10)  accD/psaI(IGS) | 3.5 | 16 | [60412-60467](#60412--60467,16,3.5,16,8)  accD/psaI(IGS) | 3.5 | All |
| **7** | 18 | [93919-94022](#93919--94022,18,5.9,17,15)  ycf2(CDS) | 5.9 | 18 | [93906-94009](#93906--94009,18,5.9,17,13)  ycf2(CDS) | 5.9 | 18 | [94446-94549](#94446--94549,18,5.9,17,15)  ycf2(CDS) | 5.9 | 18 | [93558-93661](#93558--93661,18,5.9,17,14)  ycf2(CDS) | 5.9 | All |
| **8** | 18 | [93934-94025](#93934--94025,18,5.1,18,16)  ycf2(CDS) | 5.1 | 18 | [93921-94012](#93921--94012,18,5.1,18,14)  ycf2(CDS) | 5.1 | 18 | [94461-94552](#94461--94552,18,5.1,18,16)  ycf2(CDS) | 5.1 | 18 | [93573-93664](#93573--93664,18,5.1,18,15)  ycf2(CDS) | 5.1 | All |
| **9** | 20 | [114464-114505](#114464--114505,20,2.0,21,23)  ndhF(CDS) | 2 | 20 | [114444-114485](#114444--114485,20,2.0,21,20)  ndhF(CDS) | 2 | 20 | [114987-115028](#114987--115028,20,2.0,21,22)  ndhF(CDS) | 2 | 20 | [114103-114144](#114103--114144,20,2.0,21,22)  ndhF(CDS) | 2 | All |
| **10** | 18 | [149755-149846](#149755--149846,18,5.1,18,36)  ycf2(CDS) | 5.1 | 18 | [149738-149829](#149738--149829,18,5.1,18,31)  ycf2(CDS) | 5.1 | 18 | [150016-150107](#150016--150107,18,5.1,18,34)  ycf2(CDS) | 5.1 | 18 | [149400-149491](#149400--149491,18,5.1,18,36)  ycf2(CDS) | 5.1 | All |
| **11** | 18 | [149762-149861](#149762--149861,18,5.8,17,37)  ycf2(CDS) | 5.8 | 18 | [149745-149844](#149745--149844,18,5.8,17,32)  ycf2(CDS) | 5.8 | 18 | [150023-150122](#150023--150122,18,5.8,17,35)  ycf2(CDS) | 5.8 | 18 | [149407-149506](#149407--149506,18,5.8,17,37)  ycf2(CDS) | 5.8 | All |
| **12** | 25 | [4434-4480](#4434--4480,25,2.0,25,1)  matK/rps16(IGS) | 2 | 25 | [4427-4473](#4427--4473,25,2.0,25,1)  matK/rps16(IGS) | 2 | 25 | [5075-5121](#5075--5121,25,2.0,25,1)  matK/rps16(IGS) | 2 | 25 | [4433-4479](#4433--4479,25,2.0,25,1)  matK/rps16(IGS) | 2 | All |
| **13** | 22 | [6409-6459](#6409--6459,22,2.3,23,3)  rps16/trnQ-UUG  (IGS) | 2.3 | 22 | [6397-6447](#6397--6447,22,2.3,23,3)  rps16/trnQ-UUG  (IGS) | 2.3 | 22 | [7048-7098](#7048--7098,22,2.3,23,3)  rps16/trnQ-UUG  (IGS) | 2.3 | 22 | [6401-6451](#6401--6451,22,2.3,23,3)  rps16/trnQ-UUG  (IGS) | 2.3 | All |
| **14** | 24 | [91476-91530](#91476--91530,24,2.5,22,11)  ycf2(CDS) | 2.5 | 24 | [91463-91517](#91463--91517,24,2.5,22,9)  ycf2(CDS) | 2.5 | 24 | [92003-92057](#92003--92057,24,2.5,22,11)  ycf2(CDS) | 2.5 | 24 | [91115-91169](#91115--91169,24,2.5,22,10)  ycf2(CDS) | 2.5 | All |
| **15** | 21 | [91498-91565](#91498--91565,21,3.2,21,14)  ycf2(CDS) | 3.2 | 21 | [91485-91552](#91485--91552,21,3.2,21,12)  ycf2(CDS) | 3.2 | 21 | [92025-92092](#92025--92092,21,3.2,21,14)  ycf2(CDS) | 3.2 | 21 | [91137-91204](#91137--91204,21,3.2,21,13)  ycf2(CDS) | 3.2 | All |
| **16** | 21 | [152215-152304](#152215--152304,21,4.1,22,41)  ycf2(CDS) | 4.1 | 21 | [152198-152287](#152198--152287,21,4.1,22,36)  ycf2(CDS) | 4.1 | 21 | [152476-152565](#152476--152565,21,4.1,22,39)  ycf2(CDS) | 4.1 | 21 | [151860-151949](#151860--151949,21,4.1,22,41)  ycf2(CDS) | 4.1 | All |
| **17** | 18 | [101901-101937](#101901--101937,18,2.1,18,21)  rps7/ycf15(IGS) | 2.1 | 18 | [101888-101924](#101888--101924,18,2.1,18,19)  rps7/ycf15(IGS) | 2.1 | 18 | [102429-102465](#102429--102465,18,2.1,18,21)  rps7/ycf15(IGS) | 2.1 | 18 | [101540-101576](#101540--101576,18,2.1,18,20)  rps7/ycf15(IGS) | 2.1 | All |
| **18** | 23 | [101364-101415](#101364--101415,23,2.3,24,20)  rps7/ycf15(IGS) | 2.3 | 23 | [101351-101402](#101351--101402,23,2.3,24,18)  rps7/ycf15(IGS) | 2.3 | 23 | [101892-101943](#101892--101943,23,2.3,24,20)  rps7/ycf15(IGS) | 2.3 | 23 | [101003-101054](#101003--101054,23,2.3,24,19)  rps7/ycf15(IGS)) | 2.3 | All |
| **19** | 23 | [142365-142416](#142365--142416,23,2.3,23,30)  ycf15/rps7(IGS) | 2.3 | 23 | [142348-142399](#142348--142399,23,2.3,23,25)  ycf15/rps7(IGS) | 2.3 | 23 | [142625-142676](#142625--142676,23,2.3,23,28)  ycf15/rps7(IGS) | 2.3 | 23 | [142010-142061](#142010--142061,23,2.3,23,30)  ycf15/rps7(IGS) | 2.3 | All |
| **20** | 17 | [44352-44391](#44352--44391,17,2.4,17,4)  psaA/ycf3(IGS) | 2.4 |  |  |  | 17 | [44988-45027](#44988--45027,17,2.4,17,4)  psaA/ycf3(IGS) | 2.4 |  |  |  | *CSS、*  *CIA* |
| **21** | 16 | [112707-112740](#112707--112740,16,2.1,17,22)  ycf1-5end (CDS) | 2.1 |  |  |  |  |  |  | 16 | [112346-112379](#112346--112379,16,2.1,17,21)  ycf1-5end (CDS) | 2.1 | *CSS、CWN* |
| **22** | 16 | [131040-131073](#131040--131073,16,2.1,17,27)  ycf1(CDS) | 2.1 |  |  |  |  |  |  | 16 | [130685-130718](#130685--130718,16,2.1,17,27)  ycf1(CDS) | 2.1 | *CSS、CWN* |
| **23** | 11 | [69296-69332](#69296--69332,11,3.5,11,10)  trnP-UGG/psaJ  (IGS) | 3.5 |  |  |  |  |  |  |  |  |  | *CSS* |
| **24** |  |  |  |  |  |  | 15 | [57398-57428](#57398--57428,15,2.1,15,7)  atpB/rbcL(IGS) | 2.1 |  |  |  | *CIA* |
| **25** |  |  |  |  |  |  |  |  |  | 14 | [68654-68684](#68654--68684,14,2.2,14,9)  trnW-CCA/ trnP-UGG(IGS) | 2.2 | *CWN* |
| **26** |  |  |  |  |  |  |  |  |  | 24 | [123012-123063](#123012--123063,24,2.1,24,24)  ndhA(Intron) | 2.1 | *CWN* |
| **Num** |  | CDS, 11; IGS, 12; Intron, 0 | |  | CDS, 9; IGS, 10; Intron, 0 | |  | CDS, 9; IGS, 12; Intron, 0 | |  | CDS, 11; IGS, 11; Intron, 1 | |  |
|  | Number of different locations: CDS, 40; IGS, 45; Intron, 1 | | | | | | | | | | | | |

| **Forward repeats** | | | | | | | | | | | | | **Shared** |
| --- | --- | --- | --- | --- | --- | --- | --- | --- | --- | --- | --- | --- | --- |
| ***CSS*** | | | | ***CSA*** | | | ***CIA*** | | | ***CWN*** | | |  |
| **No.** | **Len** | **Position 1** | **Position 2** | **Len** | **Position 1** | **Position 2** | **Len** | **Position 1** | **Position 2** | **Len** | **Position 1** | **Position 2** |  |
| **1** | 82 | 93929  ycf2（CDS） | 93947  ycf2（CDS） | 82 | 93916  ycf2（CDS） | 93934  ycf2（CDS） | 82 | 94456  ycf2（CDS） | 94474  ycf2（CDS） | 82 | 93568  ycf2（CDS） | 93586  ycf2（CDS） | All |
| **2** | 82 | 149750  ycf2（CDS） | 149768  ycf2（CDS） | 82 | 149733  ycf2（CDS） | 149751  ycf2（CDS） | 82 | 150011  ycf2（CDS） | 150029  ycf2（CDS） | 82 | 149395  ycf2（CDS） | 149413  ycf2（CDS） | All |
| **3** | 66 | 93941  ycf2（CDS） | 93959  ycf2（CDS） | 66 | 93928  ycf2（CDS） | 93946  ycf2（CDS） | 66 | 94468  ycf2（CDS） | 94486  ycf2（CDS） | 66 | 93580  ycf2（CDS） | 93598  ycf2（CDS） | All |
| **4** | 56 | 93951  ycf2（CDS） | 93969  ycf2（CDS） | 56 | 93938  ycf2（CDS） | 93956  ycf2（CDS） | 56 | 94478  ycf2（CDS） | 94496  ycf2（CDS） | 56 | 93590  ycf2（CDS） | 93608  ycf2（CDS） | All |
| **5** | 48 | 93941  ycf2（CDS） | 93977  ycf2（CDS） | 48 | 93928  ycf2（CDS） | 93964  ycf2（CDS） | 48 | 94468  ycf2（CDS） | 94504  ycf2（CDS） | 48 | 93580  ycf2（CDS） | 93616  ycf2（CDS） | All |
| **6** | 42 | 101012  rps7/ycf15（IGS） | 122951  ndhA(Intron) | 42 | 100999  rps7/ycf15（IGS）） | 122929  ndhA(Intron) | 42 | 101540  rps7/ycf15（IGS） | 123275  ndhI/ndhA  (IGS) | 42 | 100651  rps7/ycf15（IGS） | 122591  ndhA(Intron) | All |
| **7** | 38 | 93951  ycf2（CDS） | 93987  ycf2（CDS） | 38 | 93938  ycf2（CDS） | 93974  ycf2（CDS） | 38 | 94478  ycf2（CDS） | 94514  ycf2（CDS） | 38 | 93590  ycf2（CDS） | 93626  ycf2（CDS） | All |
| **8** | 42 | 93933  ycf2（CDS） | 93987  ycf2（CDS） | 42 | 93920  ycf2（CDS） | 93974  ycf2（CDS） | 42 | 94460  ycf2（CDS） | 94514  ycf2（CDS） | 42 | 93572  ycf2（CDS） | 93626  ycf2（CDS） | All |
| **9** | 42 | 149754  ycf2（CDS） | 149808  ycf2（CDS） | 42 | 149737  ycf2（CDS） | 149791  ycf2（CDS） | 42 | 150015  ycf2（CDS） | 150069  ycf2（CDS） | 42 | 149399  ycf2（CDS） | 149453  ycf2（CDS） | All |
| **10** | 39 | 45608  ycf3(Intron) | 101014  rps7/ycf15  (IGS) | 39 | 45576  ycf3(Intron) | 101001  rps7/ycf15  (IGS) | 39 | 46244  ycf3(Intron) | 101542  rps7/ycf15  (IGS) | 39 | 45238  ycf3(Intron) | 100653  rps7/ycf15  (IGS) | All |
| **11** | 39 | 45608  ycf3(CDS) | 122953  ndhA(Intron) | 39 | 45576  ycf3(Intron) | 122931  ndhA(Intron) | 39 | 46244  ycf3(Intron) | 123277  ndhA(Intron) | 39 | 45238  ycf3(Intron) | 122593  ndhA（CDS） | All |
| **12** | 38 | 60773  accD/psaI  (IGS) | 60789  accD/psaI  (IGS) | 38 | 60756  accD/psaI  (IGS) | 60772  accD/psaI  (IGS) | 38 | 61411  accD/psaI  (IGS) | 61427  accD/psaI  (IGS) | 38 | 60408  accD/psaI  (IGS) | 60424  accD/psaI  (IGS) | All |
| **13** | 34 | 93941  ycf2（CDS） | 93995  ycf2（CDS） | 34 | 93928  ycf2（CDS） | 93982  ycf2（CDS） | 34 | 94468  ycf2（CDS） | 94522  ycf2（CDS） | 34 | 93580  ycf2（CDS） | 93634  ycf2（CDS） | All |
| **14** | 35 | 40545  psaB(CDS) | 42769  psaA(CDS) | 35 | 40529  psaB(CDS) | 42753  psaA(CDS) | 35 | 41179  psaB(CDS) | 43403  psaA(CDS) | 35 | 40192  psaB(CDS) | 42416  psaA(CDS) | All |
| **15** | 32 | 9045  psbI/trnS-GCU  (IGS) | 37389  psbC/trnS-UGA  (IGS) | 32 | 9028  psbI/trnS-GCU  (IGS) | 37372  psbC/trnS-UGA  (IGS) | 32 | 9685  psbI/trnS-GCU  (IGS) | 38021  psbC/trnS-UGA  (IGS) | 32 | 9036  psbI/trnS-GCU  (IGS) | 37035  psbC/trnS-UGA  (IGS) | All |
| **16** | 60 | 93933  ycf2（CDS） | 93969  ycf2（CDS） | 60 | 93920  ycf2（CDS） | 93956  ycf2（CDS） |  |  |  | 60 | 93572  ycf2（CDS） | 93608  ycf2（CDS） | *CSS、CSA、CWN* |
| **17** | 60 | 149754  ycf2（CDS） | 149790  ycf2（CDS） | 60 | 149737  ycf2（CDS） | 149773  ycf2（CDS） |  |  |  | 60 | 149399  ycf2（CDS） | 149435  ycf2（CDS） | *CSS、CSA、CWN* |
| **18** | 30 | 45620  ycf3(Intron) | 101026  rps7/ycf15  (IGS) | 30 | 45588  ycf3(Intron) | 101013  rps7/ycf15  (IGS) |  |  |  | 30 | 45250  ycf3(Intron) | 100665  rps7/ycf15  (IGS) | *CSS、CSA、CWN* |
| **19** | 30 | 91493  ycf2（CDS） | 91535  ycf2（CDS） | 30 | 91480  ycf2（CDS） | 91522  ycf2（CDS） |  |  |  | 30 | 91132  ycf2（CDS） | 91174  ycf2（CDS） | *CSS、CSA、CWN* |
| **20** | 30 | 152214  ycf2（CDS） | 152256  ycf2（CDS） | 30 | 152197  ycf2（CDS） | 152239  ycf2（CDS） |  |  |  | 30 | 151859  ycf2（CDS） | 151901  ycf2（CDS） | *CSS、CSA、CWN* |
| **21** |  |  |  |  |  |  | 80 | 0  rpl2/trnH-GUG (IGS） | 151677  ycf2（CDS） |  |  |  | *CIA* |
| **22** |  |  |  |  |  |  | 59 | 366  rpl2/trnH-GUG(IGS) | 157117  rpl2/trnH-GUG(IGS) |  |  |  | *CIA* |
| **23** |  |  |  |  |  |  | 50 | 497  rpl2/trnH-GUG(IGS) | 157238  rpl2/trnH-GUG(IGS) |  |  |  | *CIA* |
| **24** |  |  |  |  |  |  | 49 | 96926  ycf15/trnL-CCA(IGS) | 147592  trnL-CAA/ycf15(IGS) |  |  |  | *CIA* |
| **25** |  |  |  |  |  |  | 41 | 91  rpl2/trnH-GUG(IGS) | 156865  rpl2（CDS） |  |  |  | *CIA* |
| **26** |  |  |  |  |  |  | 34 | 391  rpl2/trnH-GUG(IGS) | 157142  rpl2（CDS） |  |  |  | *CIA* |
| **27** |  |  |  |  |  |  | 34 | 136  rpl2/trnH-GUG(IGS) | 156908  rpl2（CDS） |  |  |  | *CIA* |
| **28** |  |  |  |  |  |  | 30 | 189  rpl2/trnH-GUG(IGS) | 156960  rpl2（CDS） |  |  |  | *CIA* |
| **Num** |  | CDS, 29; IGS, 7; Intron, 4 | |  | CDS, 28; IGS, 7; Intron,5 | |  | CDS, 25; IGS, 18; Intron, 3 | |  | CDS, 29; IGS, 7; Intron, 4 | |  |
| Number of different locations: CDS, 111; IGS, 39; Intron, 16 | | | | | | | | | | | | | |

| **Palindromic repeats** | | | | | | | | | | | | | | **Shared** |
| --- | --- | --- | --- | --- | --- | --- | --- | --- | --- | --- | --- | --- | --- | --- |
| ***CSS*** | | | | ***CSA*** | | | ***CIA*** | | | | ***CWN*** | | |  |
| **No.** | **Len** | **Position 1** | **Position 2** | **Len** | **Position 1** | **Position 2** | **Len** | **Position 1** | | **Position 2** | **Len** | **Position 1** | **Position 2** |  |
| **1** | 82 | 93929  ycf2（CDS） | 149750  ycf2（CDS） | 82 | 93916  ycf2（CDS） | 149733  ycf2（CDS） | 82 | 94456  ycf2（CDS） | | 150011  ycf2（CDS） | 82 | 93568  ycf2（CDS） | 149395  ycf2（CDS） | All |
| **2** | 82 | 93947  ycf2（CDS） | 149768  ycf2（CDS） | 82 | 93934  ycf2（CDS） | 149751  ycf2（CDS） | 82 | 94474  ycf2（CDS） | | 150029  ycf2（CDS） | 82 | 93586  ycf2（CDS） | 149413  ycf2（CDS） | All |
| **3** | 66 | 93941  ycf2（CDS） | 149754  ycf2（CDS） | 66 | 93928  ycf2（CDS） | 149737  ycf2（CDS） | 66 | 94468  ycf2（CDS） | | 150015  ycf2（CDS） | 66 | 93580  ycf2（CDS） | 149399  ycf2（CDS） | All |
| **4** | 66 | 93959  ycf2（CDS） | 149772  ycf2（CDS） | 66 | 93946  ycf2（CDS） | 149755  ycf2（CDS） | 66 | 94486  ycf2（CDS） | | 150033  ycf2（CDS） | 66 | 93598  ycf2（CDS） | 149417  ycf2（CDS） | All |
| **5** | 56 | 93951  ycf2（CDS） | 149754  ycf2（CDS） | 56 | 93938  ycf2（CDS） | 149737  ycf2（CDS） | 56 | 94478  ycf2（CDS） | | 150015  ycf2（CDS） | 56 | 93590  ycf2（CDS） | 149399  ycf2（CDS） | All |
| **6** | 56 | 93969  ycf2（CDS） | 149772  ycf2（CDS） | 56 | 93956  ycf2（CDS） | 149755  ycf2（CDS） | 56 | 94496  ycf2（CDS） | | 150033  ycf2（CDS） | 56 | 93608  ycf2（CDS） | 149417  ycf2（CDS） | All |
| **7** | 60 | 93933  ycf2（CDS） | 149750  ycf2（CDS） | 60 | 93920  ycf2（CDS） | 149733  ycf2（CDS） | 60 | 94460  ycf2（CDS） | | 150011  ycf2（CDS） | 60 | 93572  ycf2（CDS） | 149395  ycf2（CDS） | All |
| **8** | 60 | 93969  ycf2（CDS） | 149786  ycf2（CDS） | 60 | 93956  ycf2（CDS） | 149769  ycf2（CDS） | 60 | 94496  ycf2（CDS） | | 150047  ycf2（CDS） | 60 | 93608  ycf2（CDS） | 149431  ycf2（CDS） | All |
| **9** | 48 | 93941  ycf2（CDS） | 149754  ycf2（CDS） | 48 | 93928  ycf2（CDS） | 149737  ycf2（CDS） | 48 | 94468  ycf2（CDS） | | 150015  ycf2（CDS） | 48 | 93580  ycf2（CDS） | 149399  ycf2（CDS） | All |
| **10** | 48 | 93977  ycf2（CDS） | 149790  ycf2（CDS） | 48 | 93964  ycf2（CDS） | 149773  ycf2（CDS） | 48 | 94504  ycf2（CDS） | | 150051  ycf2（CDS） | 48 | 93616  ycf2（CDS） | 149435  ycf2（CDS） | All |
| **11** | 42 | 122951  ndhA(Intron) | 142725  ycf15/rps7  (IGS) | 42 | 122929  ndhA(Intron) | 142708  ycf15/rps7  (IGS) | 42 | 123275  ndhA(Intron) | | 142985  ycf15/rps7  (IGS) | 42 | 122591  ndhA(Intron) | 142370  ycf15/rps7  (IGS) | All |
| **12** | 38 | 93951  ycf2（CDS） | 149754  ycf2（CDS） | 38 | 93938  ycf2（CDS） | 149737  ycf2（CDS） | 38 | 94478  ycf2（CDS） | | 150015  ycf2（CDS） | 38 | 93590  ycf2（CDS） | 149399  ycf2（CDS） | All |
| **13** | 38 | 93987  ycf2（CDS） | 149790  ycf2（CDS） | 38 | 93974  ycf2（CDS） | 149773  ycf2（CDS） | 38 | 94514  ycf2（CDS） | | 150051  ycf2（CDS） | 38 | 93626  ycf2（CDS） | 149435  ycf2（CDS） | All |
| **14** | 42 | 93933  ycf2（CDS） | 149750  ycf2（CDS） | 42 | 93920  ycf2（CDS） | 149733  ycf2（CDS） | 42 | 94460  ycf2（CDS） | | 150011  ycf2（CDS） | 42 | 93572  ycf2（CDS） | 149395  ycf2（CDS） | All |
| **15** | 42 | 93987  ycf2（CDS） | 149804  ycf2（CDS） | 42 | 93974  ycf2（CDS） | 149787  ycf2（CDS） | 42 | 94514  ycf2（CDS） | | 150065  ycf2（CDS） | 42 | 93626  ycf2（CDS） | 149449  ycf2（CDS） | All |
| **16** | 39 | 45608  ycf3(Intron) | 142726  ycf15/rps7  (IGS) | 39 | 45576  ycf3(Intron) | 142709  ycf15/rps7  (IGS) | 39 | 46244  ycf3(Intron) | | 142986  ycf15/rps7  (IGS) | 39 | 45238  ycf3(Intron) | 142371  ycf15/rps7  (IGS) | All |
| **17** | 30 | 9047  psbI/trnS-GCU  (IGS) | 47321  trnS-GGA  (CDS) | 30 | 9030  psbI/trnS-GCU  (IGS) | 47290  trnS-GGA  (CDS) | 30 | 9687  psbI/trnS-GCU  (IGS) | | 47957  trnS-GGA(CDS) | 30 | 9038  psbI/trnS-GCU  (IGS) | 46944  trnS-GGA  (CDS) | All |
| **18** | 34 | 93941  ycf2（CDS） | 149750  ycf2（CDS） | 34 | 93928  ycf2（CDS） | 149733  ycf2（CDS） | 34 | 94468  ycf2（CDS） | | 150011  ycf2（CDS） | 34 | 93580  ycf2（CDS） | 149395  ycf2（CDS） | All |
| **19** | 34 | 93995  ycf2（CDS） | 149804  ycf2（CDS） | 34 | 93982  ycf2（CDS） | 149787  ycf2（CDS） | 34 | 94522  ycf2（CDS） | | 150065  ycf2（CDS） | 34 | 93634  ycf2（CDS） | 149449  ycf2（CDS） | All |
| **20** | 30 | 37391  psbC/trnS-UGA  (IGS) | 47321  trnS-GGA  （CDS） | 30 | 37374  psbC/trnS-UGA  (IGS) | 47290  trnS-GGA  （CDS） | 30 | 38023  psbC/trnS-UGA(IGS) | | 47957  trnS-GGA（CDS） | 30 | 37037  psbC/trnS-UGA  (IGS) | 46944  trnS-GGA  （CDS） | All |
| **21** | 30 | 45620  ycf3(Intron) | 142723  ycf15/rps7  (IGS) | 30 | 45588  ycf3(Intron) | 142706  ycf15/rps7  (IGS) | 30 | 46256  ycf3(Intron) | | 142983  ycf15/rps7  (IGS) | 30 | 45250  ycf3(Intron) | 142368  ycf15/rps7  (IGS) | All |
| **22** | 30 | 91493  ycf2（CDS） | 152214  ycf2（CDS） | 30 | 91480  ycf2（CDS） | 152197  ycf2（CDS） | 30 | 92020  ycf2（CDS） | | 152475  ycf2（CDS） | 30 | 91132  ycf2（CDS） | 151859  ycf2（CDS） | All |
| **23** | 30 | 91535  ycf2（CDS） | 152256  ycf2（CDS） | 30 | 91522  ycf2（CDS） | 152239  ycf2（CDS） | 30 | 92062  ycf2（CDS） | | 152517  ycf2（CDS） | 30 | 91174  ycf2（CDS） | 151901  ycf2（CDS） | All |
| **24** |  |  |  |  |  |  | 80 | 0  rpl2/trnH-GUG(IGS) | | 92810  ycf2（CDS） |  |  |  | *CIA* |
| **25** |  |  |  |  |  |  | 59 | 366  rpl2/trnH-GUG(IGS) | | 87391  rpl2（CDS） |  |  |  | *CIA* |
| **26** |  |  |  |  |  |  | 50 | 497  rpl2/trnH-GUG(IGS) | | 87279  rps19/rpl2(IGS) |  |  |  | *CIA* |
| **27** |  |  |  |  |  |  | 41 | 91  rpl2/trnH-GUG(IGS) | | 87661  rpl2（CDS） |  |  |  | *CIA* |
| **28** |  |  |  |  |  |  | 34 | 391  rpl2/trnH-GUG(IGS) | | 87391  rpl2（CDS） |  |  |  | *CIA* |
| **29** |  |  |  |  |  |  | 30 | 113245  ycf1（CDS） | | 131294  ycf1（CDS） |  |  |  | *CIA* |
| **30** |  |  |  |  |  |  | 34 | 136  rpl2/trnH-GUG(IGS) | | 87625  rpl2（CDS） |  |  |  | *CIA* |
| **31** |  |  |  |  |  |  | 30 | 189  rpl2/trnH-GUG(IGS) | | 87577  rpl2（CDS） |  |  |  | *CIA* |
| **32** |  |  |  |  |  |  | 31 | 77402  psbT/psbN(IGS) | | 77421  psbT/psbN(IGS) |  |  |  | *CIA* |
| Num |  | CDS,38; IGS, 5; Intron, 3 | |  | CDS:38; IGS:5; Intron:3 | |  | | CDS:46; IGS:15; Intron:3 | |  | CDS:38; IGS:5; Intron:3 | |  |
| Number of different locations: CDS, 160; IGS, 30; Intron, 12 | | | | | | | | | | | | | | |

Total number of different locations in all repeats: CDS: 311; IGS: 114; Intron: 29.

*CWN*: ‘Wuyi narcissus’ cultivar of *C. sinensis* var. *sinensis* (natural triploid Chinary type tea); *CSS*: *C. sinensis* var. *sinensis* (diploid Chinary type tea); *CSA*: *C. sinensis* var. *assamica* (diploid Chinese Assamica type tea); *CIA*: *C. sinensis* var. *assamica* (diploid Indian Assamica type tea).
