# Supplementary material for: Comparative chloroplast genomes: insights into the evolution of the chloroplast genome of Camellia sinensis and the phylogeny of Camellia
Source: BMC Genomics. 2021 Feb 26;22:138. doi: 10.1186/s12864-021-07427-2 (PMC7912895; doi:10.1186/s12864-021-07427-2)
Supplement: Supplementary file 10 — Additional file 10: Supplementary Tab. S4. Distribution of indel in four chloroplast genomes. CWN: ‘Wuyi narcissus’ cultivar of C. sinensis var. sinensis (natural triploid Chinary type tea); CSS: C. sinensis var. sinensis (diploid Chinary type tea); CSA: C. sinensis var. assamica (diploid Chinese Assamica type tea); CIA: C. sinensis var. assamica (diploid Indian Assamica type tea). [file 12864_2021_7427_MOESM10_ESM.docx]

**Supplementary Tab.S4 Distribution of Indel in four chloroplast genomes.**

| Indel Len | Num | Region | | | Species |
| --- | --- | --- | --- | --- | --- |
|  |  | CDS | Intron | IGS | (Sequence deletion) |
| 1 | 1 | trnH-GUG |  |  | *CIA* |
|  | 2 | trnH-GUG |  |  | *CIA* |
|  | 3 | trnH-GUG |  |  | *CIA* |
|  | 4 | trnH-GUG |  |  | *CIA* |
|  | 5 |  |  | trnH-GUG/psbA | *CIA* |
|  | 6 |  |  | trnH-GUG/psbA | *CIA* |
|  | 7 |  |  | trnH-GUG/psbA | *CSS、CSA、CWN* |
|  | 8 |  |  | trnH-GUG/psbA | *CSS、CSA、CWN* |
|  | 9 |  |  | trnH-GUG/psbA | *CIA* |
|  | 10 |  |  | trnH-GUG/psbA | *CIA* |
|  | 11 | psbA |  |  | *CSS、CSA、CWN* |
|  | 12 |  | rps16 |  | *CWN* |
|  | 13 |  |  | rps16/trnQ-UUG | *CSA、CIA* |
|  | 14 |  |  | rps16/trnQ-UUG | *CSS、CSA、CWN* |
|  | 15 | psbI |  |  | *CWN* |
|  | 16 |  |  | trnS-GCU/trnG-GCC | *CSS、CWN* |
|  | 17 |  |  | atpH/aptI | *CSA、CIA、CWN* |
|  | 18 |  |  | trnE-UUC/trnT-GGU | *CSA* |
|  | 19 |  |  | psbC/trnS-UGA | *CSA、CIA* |
|  | 20 |  |  | trnG-UCC/trnM-CAU | *CSS、CIA* |
|  | 21 |  | ycf3 |  | *CSS、CIA、CWN* |
|  | 22 | trnL-UAA |  |  | *CSA、CWN* |
|  | 23 | ndhK |  |  | *CSS、CSA、CIA* |
|  | 24 |  |  | ndhK/trnV-UAC | *CIA、CWN* |
|  | 25 |  |  | atpB/rbcL | *CWN* |
|  | 26 |  |  | accD/psaI | *CWN* |
|  | 27 |  |  | ycf4/cemA | *CSS、CIA、CWN* |
|  | 28 |  |  | petA/psbJ | *CSS、CSA、CWN* |
|  | 29 |  |  | petA/psbJ | *CWN* |
|  | 30 |  |  | petA/psbJ | *CSS、CIA、CWN* |
|  | 31 | psbL |  |  | *CSS、CIA、CWN* |
|  | 32 |  |  | rps18/rpl20 | *CSS、CIA、CWN* |
|  | 33 | clpP |  |  | *CSA、CWN* |
|  | 34 |  |  | clpP/psbB | *CSS、CIA、CWN* |
|  | 35 |  |  | psbN/psbH | *CSS、CSA、CWN* |
|  | 36 |  |  | psbN/psbH | *CSS、CSA、CWN* |
|  | 37 |  |  | petD/rpoA | *CSS、CSA、CWN* |
|  | 38 |  |  | rpl16/rps3 | *CSS、CSA、CWN* |
|  | 39 |  |  | ycf15/trnL-CAA | *CSS、CSA、CWN* |
|  | 40 |  | trnI-GAU |  | *CSS、CIA、CWN* |
|  | 41 | 23SrRNA |  |  | *CSS、CSA、CWN* |
|  | 42 |  |  | ndhF/rpl32 | *CWN* |
|  | 43 |  |  | rpl32/trnL-UAG | *CSA* |
|  | 44 |  | ndhA |  | *CWN* |
|  | 45 | ycf1 |  |  | *CIA* |
|  | 46 | ycf1 |  |  | *CIA* |
|  | 47 | 23SrRNA |  |  | *CSS、CSA、CWN* |
| 2 | 1 |  |  | trnH-GUG/psbA | *CIA* |
|  | 2 |  |  | trnH-GUG/psbA | *CIA* |
|  | 3 |  |  | atpA/atpF | *CSS、CSA、CIA* |
|  | 4 |  |  | petN/psbM | *CSA、CIA* |
|  | 5 |  |  | trnT-GGU/psbC | *CSA、CIA、CWN* |
|  | 6 |  |  | trnT-GGU/psbC | *CSA、CIA、CWN* |
|  | 7 |  |  | trnS-UGA/psbZ | *CSS、CSA、CWN* |
|  | 8 |  | trnV-UAC |  | *CSS、CSA、CWN* |
|  | 9 |  |  | atpB/rbcL | *CSS、CIA* |
|  | 10 |  | clpP |  | *CSS、CIA* |
|  | 11 |  |  | psbN/psbH | *CIA* |
|  | 12 |  |  | psbN/psbH | *CSS、CSA、CWN* |
|  | 13 |  |  | rps8/rpl14 | *CIA、CWN* |
|  | 14 |  |  | rpl14/rpl16 | *CSS、CIA* |
|  | 15 |  |  | rpl32/trnL-UAG | *CSA* |
|  | 16 |  |  | rpl32/trnL-UAG | *CIA* |
| 3 | 1 |  |  | atpH/aptI | *CSS、CIA、CWN* |
|  | 2 |  |  | trnT-UGU/trnL-UAA | *CSA* |
| 4 | 1 |  |  | trnH-GUG/psbA | *CSS、CSA、CWN* |
|  | 2 |  |  | trnS-GCU/trnG-GCC | *CSS、CIA、CWN* |
|  | 3 |  |  | psbB/psbT | *CIA* |
|  | 4 | ycf1 |  |  | *CIA* |
| 5 | 1 |  |  | trnK-UUU/rps16 | *CSA* |
|  | 2 |  |  | rps16/trnQ-UUG | *CSA* |
|  | 3 |  |  | ndhC/trnV-UAC | *CSS、CSA、CIA* |
|  | 4 |  |  | trnP-UGG/psaJ | *CSA、CIA、CWN* |
|  | 5 |  |  | psbT/psbN | *CSA、CIA、CWN* |
|  | 6 |  |  | ndhF/rpl32 | *CIA* |
| 6 | 1 | ycf1-5end |  |  | *CIA* |
|  | 2 | ycf1 |  |  | *CSS、CIA、CWN* |
| 7 | 1 |  | rps16 |  | *CWN* |
|  | 2 |  |  | rpoB/trnC-GCA | *CWN* |
|  | 3 |  |  | ycf3/trnS-GGA | *CWN* |
|  | 4 |  |  | ndhF/rpl32 | *CIA* |
| 8 | 1 |  |  | 4.5SrRNA/5SrRNA | *CSA* |
|  | 2 | ndhI |  |  | *CIA* |
|  | 3 | ycf1 |  |  | *CIA* |
|  | 4 |  |  | 5SrRNA/4.5SrRNA | *CIA* |
| 10 | 1 |  |  | trnT-UGU/trnL-UAA | *CSS、CIA* |
| 11 | 1 |  |  | trnH-GUG/psbA | *CSS、CSA、CWN* |
| 12 | 1 |  |  | psaA/ycf3 | *CSS、CSA、CWN* |
|  | 2 |  |  | ndhG/ndhI | *CIA* |
| 13 | 1 |  |  | ndhI/ndhA | *CIA* |
| 14 | 1 |  |  | trnW-CCA/trnP-UGG | *CSS、CSA、CIA* |
| 17 | 1 |  |  | psaA/ycf3 | *CSA、CWN* |
| 25 | 1 |  |  | rp12/trnH-GUG | *CIA* |
| 33 | 1 |  |  | ccsA/ndhD | *CIA* |
| 51 | 1 | ycf1 |  |  | *CIA* |
| 53 | 1 | ndhD |  |  | *CIA* |
| 66 | 1 | ndhD |  |  | *CIA* |
| 107 | 1 | rps19 |  |  | *CIA* |
| 335 | 1 |  |  | trnE-UUC/trnT-GGU | *CWN* |
| 637 | 1 |  |  | rp12/trnH-GUG | *CSS、CSA、CWN* |
| Total | 100 | 23 | 7 | 70 |  |

*CWN*: ‘Wuyi narcissus’ cultivar of *C. sinensis* var. *sinensis* (natural triploid Chinary type tea); *CSS*: *C. sinensis* var. *sinensis* (diploid Chinary type tea); *CSA*: *C. sinensis* var. *assamica* (diploid Chinese Assamica type tea); *CIA*: *C. sinensis* var. *assamica* (diploid Indian Assamica type tea).
