# Supplementary material for: Comparative chloroplast genomes: insights into the evolution of the chloroplast genome of Camellia sinensis and the phylogeny of Camellia
Source: BMC Genomics. 2021 Feb 26;22:138. doi: 10.1186/s12864-021-07427-2 (PMC7912895; doi:10.1186/s12864-021-07427-2)
Supplement: Supplementary file 11 — Additional file 11: Supplementary Tab. S5. Two hundred ninety two different cultivars of Camellia sinensis in China adopted for SCAR analysis in this study. Two hundred ninety two different cultivars represented the majority of Camellia sinensis varieties from 14 provinces in China. [file 12864_2021_7427_MOESM11_ESM.docx]

**Supplementary Tab. S5 292 different cultivars of *Camellia sinensis* in China adopted for** **SCAR analysis in this study.**

| **No.** | **Province** | **Variety** | **No.** | **Province** | **Variety** | **No.** | **Province** | **Variety** | **No.** | **Province** | **Variety** | **No.** | **Province** | **Variety** |
| --- | --- | --- | --- | --- | --- | --- | --- | --- | --- | --- | --- | --- | --- | --- |
| 1 | Fujian | Zgf | 61 | Fujian | Jys | 121 | Guandong | Zlx | 181 | Hunan | Amz | 241 | Sichuan | Glnpc |
| 2 | Fujian | Jlh | 62 | Fujian | Jmg | 122 | Guandong | Tfh | 182 | Hunan | Djy | 242 | Sichuan | Hq-1 |
| 3 | Fujian | Zbh | 63 | Fujian | Xtm | 123 | Guandong | Jmx | 183 | Hunan | 21-1 | 243 | Sichuan | Tfh-1 |
| 4 | Fujian | Xpcbl | 64 | Fujian | Llx | 124 | Guandong | Sz | 184 | Hunan | 53-34 | 244 | Sichuan | Nj-4 |
| 5 | Fujian | Zmd | 65 | Fujian | 0317-A | 125 | Guandong | Xr | 185 | Hunan | Bhz | 245 | Sichuan | Yz-1 |
| 6 | Fujian | Fy-6 | 66 | Fujian | 510 | 126 | Guandong | Gx | 186 | Hunan | Bjhjc-1 | 246 | Sichuan | C-5 |
| 7 | Fujian | Fddhc | 67 | Fujian | Yql | 127 | Guandong | Wydc | 187 | Hunan | Bxz | 247 | Sichuan | Zbj-1 |
| 8 | Fujian | Fddbc | 68 | Fujian | Hjp | 128 | Guandong | Ysdc | 188 | Hunan | Yl | 248 | Sichuan | C-4 |
| 9 | Fujian | Fy-7 | 69 | Fujian | P101 | 129 | Guandong | LTdc | 189 | Hunan | Fh | 249 | Sichuan | Cm-217 |
| 10 | Fujian | Fadbc | 70 | Fujian | Sjg | 130 | Guandong | Jhx | 190 | Hunan | Ys | 250 | Sichuan | CH-2 |
| 11 | Fujian | Byql | 71 | Fujian | 0306C | 131 | Guandong | Mlx | 191 | Hunan | Jbh | 251 | Sichuan | Zy |
| 12 | Fujian | Dhp | 72 | Fujian | 0318A | 132 | Guandong | Zly | 192 | Hunan | Xxh-21-3 | 252 | Sichuan | Ch-1 |
| 13 | Fujian | Rg | 73 | Fujian | 0318D | 133 | Guandong | Ysc | 193 | Hunan | Xtdc | 253 | Sichuan | Ms-23 |
| 14 | Fujian | Tmqz | 74 | Fujian | 0318E | 134 | Guandong | Njx | 194 | Hunan | Jy | 254 | Sichuan | Bsz |
| 15 | Fujian | Cl | 75 | Fujian | 0318F | 135 | Guandong | Zy | 195 | Hunan | Xtwdc | 255 | Sichuan | Bc-2 |
| 16 | Fujian | Hd | 76 | Fujian | 0205D | 136 | Guandong | Jlx | 196 | Hunan | Yl | 256 | Sichuan | Wmz |
| 17 | Fujian | Bd | 77 | Fujian | 3056 | 137 | Guandong | Syzhzx | 197 | Hunan | Gqz-4 | 257 | Chongqing | Bytz |
| 18 | Fujian | Bsx | 78 | Fujian | 0331E | 138 | Guandong | Wddw | 198 | Hunan | Ncdc | 258 | Chongqing | Sy-401 |
| 19 | Fujian | Jlp | 79 | Fujian | 0209-10 | 139 | Guandong | Dwy | 199 | Hunan | Ywl | 259 | Chongqing | Sy-1 |
| 20 | Fujian | Rx | 80 | Fujian | Zhdbc | 140 | Guandong | Hy-12 | 200 | Hunan | Gqz-6 | 260 | Chongqing | Sy-2 |
| 21 | Fujian | Mz | 81 | Fujian | Fy-595 | 141 | Taiwan | Qxwl | 201 | Hunan | Jn-1 | 261 | Chongqing | Jj |
| 22 | Fujian | Mk-1 | 82 | Fujian | Fy11-35 | 142 | Taiwan | Cy | 202 | Hunan | Ff-20 | 262 | Chongqing | Sy-906 |
| 23 | Fujian | Bjg-Y | 83 | Fujian | Fy-20 | 143 | Taiwan | Qxdm | 203 | Hunan | Gqz-1 | 263 | Chongqing | Y-2 |
| 24 | Fujian | Hgy | 84 | Fujian | Fy-10 | 144 | Taiwan | Jx | 204 | Hunan | Tdy | 264 | Chongqing | Sy-3 |
| 25 | Fujian | Jmd | 85 | Fujian | Cyql | 145 | Zhejiang | Ljcy | 205 | Hunan | Jhkc | 265 | Chongqing | Sy-808 |
| 26 | Fujian | 0325-A | 86 | Fujian | Fy-591 | 146 | Zhejiang | By-1 | 206 | Hunan | Yyc | 266 | Chongqing | Nj-2 |
| 27 | Fujian | 0314-C | 87 | Fujian | Yc | 147 | Zhejiang | Zc-108 | 207 | Hunan | Hldyz | 267 | Chongqing | Sy-703 |
| 28 | Fujian | T2 | 88 | Fujian | Qxql | 148 | Zhejiang | Wlz | 208 | Hunan | Zzc | 268 | Chongqing | Y-1 |
| 29 | Fujian | Lyqq | 89 | Fujian | Dh | 149 | Zhejiang | Ys | 209 | Hunan | Xfc | 269 | Hubei | E-5 |
| 30 | Fujian | 0214-1 | 90 | Fujian | Zgy | 150 | Zhejiang | Lj-43 | 210 | Hunan | Xbl-2 | 270 | Hubei | E-12 |
| 31 | Fujian | Hmg | 91 | Fujian | Qgx | 151 | Zhejiang | Hjy | 211 | Hunan | Mf | 271 | Hubei | E-11 |
| 32 | Fujian | 0331-H | 92 | Fujian | Zfc | 152 | Zhejiang | By | 212 | Hunan | Xx-1 | 272 | Hubei | E-1 |
| 33 | Fujian | Bty | 93 | Fujian | Dywl | 153 | Zhejiang | Zc-302 | 213 | Hunan | Xh-3 | 273 | Hubei | E-10 |
| 34 | Fujian | 0205-C | 94 | Fujian | Dg | 154 | Zhejiang | Qnx | 214 | Sichuan | C-9 | 274 | Yunnan | Qtpz |
| 35 | Fujian | 0206-A | 95 | Fujian | 0319 | 155 | Zhejiang | Pytzc | 215 | Sichuan | Msbh-131 | 275 | Yunnan | Yk-10 |
| 36 | Fujian | 0312-B | 96 | Fujian | Ajwl | 156 | Zhejiang | Zn-113 | 216 | Sichuan | C-2 | 276 | Yunnan | Y63-2 |
| 37 | Fujian | 0331-I | 97 | Fujian | Bxc | 157 | Zhejiang | Zn-702 | 217 | Sichuan | Tzc | 277 | Yunnan | Nnsdcs-P |
| 38 | Fujian | 0326-A | 98 | Fujian | Bmd | 158 | Zhejiang | Zc-112 | 218 | Sichuan | Tfc-28 | 278 | Yunnan | Zj |
| 39 | Fujian | 0212-12 | 99 | Fujian | Zyh | 159 | Zhejiang | Jf | 219 | Sichuan | C-3 | 279 | Yunnan | Nndsc |
| 40 | Fujian | 0326-B | 100 | Fujian | Xhp | 160 | Zhejiang | Cf | 220 | Sichuan | Ms-4 | 280 | Yunnan | Ydz |
| 41 | Fujian | Rm | 101 | Fujian | Ljm | 161 | Zhejiang | Jlc | 221 | Sichuan | Mbl | 281 | Anhui | Ah-3 |
| 42 | Fujian | Qq | 102 | Fujian | Bs | 162 | Zhejiang | Zn-901 | 222 | Sichuan | Ms-9 | 282 | Anhui | Hz-2 |
| 43 | Fujian | Zty | 103 | Fujian | Tgy | 163 | Zhejiang | Zn-701 | 223 | Sichuan | Ms-11 | 283 | Anhui | Szc |
| 44 | Fujian | 0331-G | 104 | Fujian | Mx | 164 | Zhejiang | Zn-121 | 224 | Sichuan | Emwc | 284 | Anhui | Hsz |
| 45 | Fujian | 0331-F | 105 | Fujian | Sl | 165 | Zhejiang | Zn-902 | 225 | Sichuan | Cqppc | 285 | Anhui | W-91 |
| 46 | Fujian | Yjlx | 106 | Fujian | Zgj | 166 | Zhejiang | Zn-12 | 226 | Sichuan | Tfc-11 | 286 | Guizhou | Qm-809 |
| 47 | Fujian | Yzl | 107 | Fujian | Bjg | 167 | Zhejiang | Zn-117 | 227 | Sichuan | Sk-3 | 287 | Guizhou | Mjl |
| 48 | Fujian | Tlh | 108 | Fujian | Hd | 168 | Zhejiang | Sgc | 228 | Sichuan | Sk-36 | 288 | Guizhou | Tx03-10 |
| 49 | Fujian | Qs | 109 | Fujian | Hg | 169 | Zhejiang | Zc-102 | 229 | Sichuan | Cnhyz | 289 | Guizhou | Zs |
| 50 | Fujian | Zmg | 110 | Fujian | Lg | 170 | Zhejiang | Jg | 230 | Sichuan | Sk-1 | 290 | Jiangxi | Dyl |
| 51 | Fujian | Cg | 111 | Fujian | Gg | 171 | Zhejiang | Qnx-2 | 231 | Sichuan | Msx-311 | 291 | Shanxi | X-1 |
| 52 | Fujian | Jfh | 112 | Fujian | Sjc | 172 | Zhejiang | Hjy | 232 | Sichuan | Tz-213 | 292 | Henan | Xy-10 |
| 53 | Fujian | Fs | 113 | Guandong | Zlx | 173 | Zhejiang | Zh-1 | 233 | Sichuan | Xpz79-38-9 |  |  |  |
| 54 | Fujian | Jmh | 114 | Guandong | Bxx | 174 | Zhejiang | Nb-1 | 234 | Sichuan | Cm28 |  |  |  |
| 55 | Fujian | Gzj | 115 | Guandong | Lxw | 175 | Zhejiang | Yjx | 235 | Sichuan | Nj-1 |  |  |  |
| 56 | Fujian | Qx | 116 | Guandong | Hd | 176 | Hunan | Zyq | 236 | Sichuan | Zbj-5 |  |  |  |
| 57 | Fujian | Bmh | 117 | Guandong | Fhkc | 177 | Hunan | Hjc-2 | 237 | Sichuan | Cp71-1 |  |  |  |
| 58 | Fujian | Rzwl | 118 | Guandong | Cm | 178 | Hunan | Xbl | 238 | Sichuan | Xsz |  |  |  |
| 59 | Fujian | Yl | 119 | Guandong | Tcx | 179 | Hunan | Hjc-1 | 239 | Sichuan | Em-1 |  |  |  |
| 60 | Fujian | Ylx | 120 | Guandong | Fhsx | 180 | Hunan | Tydy | 240 | Sichuan | Jhxc |  |  |  |

292 different cultivars represented the majority of *Camellia* *sinensis* varieties from 14 provinces in China.
