# Supplementary material for: Comparative chloroplast genomes: insights into the evolution of the chloroplast genome of Camellia sinensis and the phylogeny of Camellia
Source: BMC Genomics. 2021 Feb 26;22:138. doi: 10.1186/s12864-021-07427-2 (PMC7912895; doi:10.1186/s12864-021-07427-2)
Supplement: Supplementary file 12 — Additional file 12: Supplementary Tab. S6. Chloroplast genomes of species adopted in this study. No. 1–37: 37 Camellia species; No. 38–40: 3 Polyspora species; No. 41–43: 3 Panax species; No. 44: Outgroup (Aralia undulata); No. 3, 13 and 17: 3 Camellia sinensis chloroplast reference genomes. [file 12864_2021_7427_MOESM12_ESM.docx]

**Supplementary Tab. S6 Chloroplast genomes of species adopted in this study.**

| **No.** | **Classification/species** | **Accession number** |
| --- | --- | --- |
| 1 | *Camellia tachangensis* | KY406759 |
| 2 | *Camellia danzaiensis* | NC_022460 |
| 3 | *Camellia sinensis* var. *assamica* (Indian Assamica type tea) | MH460639 |
| 4 | *Camellia sinensis* var. *sinensis* (common tea cultivar) | NC_020019 |
| 5 | *Camellia oleifera* | NC_023084 |
| 6 | *Camellia ptilophylla* | NC_038198 |
| 7 | *Camellia grandibracteata* | KJ806274 |
| 8 | *Camellia leptophylla* | KJ806275 |
| 9 | *Camellia sinensis* var. *dehungensis* | KJ806279 |
| 10 | *Camellia sinensis* var. *pubilimba* | KJ806280 |
| 11 | *Camellia taliensis* | NC_022264 |
| 12 | *Camellia gymnogyna* | NC_039626 |
| 13 | *Camellia sinensis* var. *sinensis* (Chinary type tea) | KJ806281 |
| 14 | *Camellia sinensis* var. *sinensis* (Anhua cultivar) | MH042531 |
| 15 | *Camellia granthamiana* | NC_038181 |
| 16 | *Camellia sinensis* var. *sinensis* (Longjing 43 cultivar) | KF562708 |
| 17 | *Camellia sinensis* var. *assamica* (Chinese Assamica type tea) | MH019307 |
| 18 | *Camellia huana* | KY626040 |
| 19 | *Camellia liberofilamenta* | KY626041 |
| 20 | *Camellia luteoflora* | KY626042 |
| 21 | *Camellia cuspidata* | NC_022459 |
| 22 | *Camellia szechuanensis* | NC_035651 |
| 23 | *Camellia chekiangoleosa* | NC_037472 |
| 24 | *Camellia nitidissima* | NC_039645 |
| 25 | *Camellia renshanxiangiae* | NC_041672 |
| 26 | *Camellia petelotii* | KJ806276 |
| 27 | *Camellia pubicosta* | KJ806277 |
| 28 | *Camellia reticulata* | KJ806278 |
| 29 | *Camellia azalea* | NC_035574 |
| 30 | *Camellia japonica* | NC_036830 |
| 31 | *Camellia mairei* | NC_035688 |
| 32 | *Camellia elongata* | NC_035652 |
| 33 | *Camellia crapnelliana* | NC_024541 |
| 34 | *Camellia yunnanensis* | NC_022463 |
| 35 | *Camellia pitardii* | NC_022462 |
| 36 | *Camellia impressinervis* | NC_022461 |
| 37 | *Camellia* *sinensis* var. *sinensis* (‘Wuyi narcissus’ cultivar) | MT612435 |
| 38 | *Polyspora longicarpa* | KY406768 |
| 39 | *Polyspora hainanensis* | KY406776 |
| 40 | *Polyspora speciosa* | KY406754 |
| 41 | *Panax ginseng* | KF431956 |
| 42 | *Panax notoginseng* | KJ566590 |
| 43 | *Panax quinquefolius* | NC_027456 |
| 44 | *Aralia undulata* | KC456163 |

No. 1-37: 37 *Camellia* species; No. 38-40: 3 *Polyspora* species; No. 41-43: 3 *Panax* species; No. 44: Outgroup (*Aralia undulata*); No. 3, 13 and 17: 3 *Camellia* *sinensis* chloroplast reference genomes.
