# Supplementary material for: Comparative chloroplast genomes: insights into the evolution of the chloroplast genome of Camellia sinensis and the phylogeny of Camellia
Source: BMC Genomics. 2021 Feb 26;22:138. doi: 10.1186/s12864-021-07427-2 (PMC7912895; doi:10.1186/s12864-021-07427-2)
Supplement: Supplementary file 13 — Additional file 13: Supplementary Tab. S7. Variable sites in 37 Camellia chloroplast genomes and the best-fitting models for phylogenetic analysis. [file 12864_2021_7427_MOESM13_ESM.docx]

**Supplementary Tab. S7 Variable sites in 37 *Camellia* chloroplast genomes and the best-fitting models for phylogenetic analysis.**

|  | Number of  sites | Variable sites | | Informative sites | | Nucleotide  Diversity | Best-fitting models |
| --- | --- | --- | --- | --- | --- | --- | --- |
|  |  | Numbers | % | Numbers | % |  |  |
| Complete  cp genome | 158787 | 2067 | 1.30 | 726 | 0.46 | 0.00136 | GTR+G |
| LSC | 88182 | 1362 | 1.54 | 494 | 0.56 | 0.00164 | GTR+I+G |
| SSC | 18511 | 430 | 2.32 | 133 | 0.72 | 0.00238 | GTR+I+G |
| IR | 26154 | 130 | 0.50 | 44 | 0.17 | 0.00052 | GTR+I+G |
| PCGs | 86959 | 1054 | 1.21 | 287 | 0.33 | 0.00121 | GTR+I+G |
| Non- PCGs | 83808 | 1367 | 1.65 | 372 | 0.45 | 0.00218 | GTR+I+G |
