# Supplementary material for: Comparative chloroplast genomes: insights into the evolution of the chloroplast genome of Camellia sinensis and the phylogeny of Camellia
Source: BMC Genomics. 2021 Feb 26;22:138. doi: 10.1186/s12864-021-07427-2 (PMC7912895; doi:10.1186/s12864-021-07427-2)
Supplement: Supplementary file 14 — Additional file 14: Supplementary Tab. S8. Primers used for assembly and junction verification of the chloroplast genome. [file 12864_2021_7427_MOESM14_ESM.docx]

**Supplementary Tab. S8. Primers used for assembly and junction verification of the chloroplast genome.**

| **No.** | **Region** | **Position** | **Prime sequence** | **Length (bp)** |
| --- | --- | --- | --- | --- |
| 1 | IR-SSC | ycf1-5end/ndhF | F: CAGCATTCAATGTGGATTCCTG  R: TCCTCTACTTGTATTGGTCCTA | 1342 |
| 2 | SSC-IR | ycf1 | F: CGGAATCCATTCTTCCTTGT  R: CAATGTTAGC CAGATTAGTC | 1322 |
| 3 | IR-LSC | rpl2/psbA | F: CCTTAGGCACGGCCATACAT  R: GGCCTGTAGTAGGTATCTGG | 1210 |
| 4 | LSC-IR | rps3/rpl2 | F: CCCGCTACTATCCATATATA  R: GCGTTGGCTAGGTAAGCGTC | 1035 |
| 5 | SSC | trnS-GCU/  trnG-GCC | F: CGAACCCTCGGTACAAATAAC  R: GGGGTTAATAGAACGAATCGC | 786 |
| 6 | LSC | petD | F: TCCTATGCATTGACAGGATCT  R: GGTTGTCGCGACTGGACGAC | 852 |
| 7 | LSC | trnE-UUC/  trnT-GGU | F: TCCTGAACCACTAGACGATG  R: ATGGCGTTACTCTACCACTG | 829 |
| 8 | LSC | clpP/psbB | F: GATCTAATTGCACTTCACGC  R: GTTACAGTCCCTCCTGTGAT | 911 |
| 9 | SSC | ndhA | F: ACCAATGGAATTCTGTCTGC  R: CATCATTCGAGTTGATGAAC | 813 |
| 10 | IR | ycf1-5end | F: GGGACAGCTCATGATGTTCA  R: TGATTCCCATCCAGATAAGA | 812 |
